# Genetic landscape of 6089 inherited retinal dystrophies affected cases in Spain and their therapeutic and extended epidemiological implications

**DOI:** 10.1038/s41598-021-81093-y

**Published:** 2021-01-15

**Authors:** Irene Perea-Romero, Gema Gordo, Ionut F. Iancu, Marta Del Pozo-Valero, Berta Almoguera, Fiona Blanco-Kelly, Ester Carreño, Belen Jimenez-Rolando, Rosario Lopez-Rodriguez, Isabel Lorda-Sanchez, Inmaculada Martin-Merida, Lucia Pérez de Ayala, Rosa Riveiro-Alvarez, Elvira Rodriguez-Pinilla, Saoud Tahsin-Swafiri, Maria J. Trujillo-Tiebas, The ESRETNET Study Group, Ana Bustamante-Aragones, Rocio Cardero-Merlo, Ruth Fernandez-Sanchez, Jesus Gallego-Merlo, Ines Garcia-Vara, Ascension Gimenez-Pardo, Laura Horcajada-Burgos, Fernando Infantes-Barbero, Esther Lantero, Miguel A. Lopez-Martinez, Andrea Martinez-Ramas, Lorena Ondo, Marta Rodriguez de Alba, Carolina Sanchez-Jimeno, Camilo Velez-Monsalve, Cristina Villaverde, Olga Zurita, Domingo Aguilera-Garcia, Jana Aguirre-Lamban, Ana Arteche, Diego Cantalapiedra, Patricia Fernandez-San Jose, Liliana Galbis-Martinez, Maria Garcia-Hoyos, Carlos Lombardia, Maria I. Lopez-Molina, Raquel Perez-Carro, Luciana R. J. Da Silva, Carmen Ramos, Rocio Sanchez-Alcudia, Iker Sanchez-Navarro, Sorina D. Tatu, Elena Vallespin, Elena Aller, Sara Bernal, Maria J. Gamundi, Gema Garcia-Garcia, Inmaculada Hernan, Teresa Jaijo, Guillermo Antiñolo, Montserrat Baiget, Miguel Carballo, Jose M. Millan, Diana Valverde, The ERDC Study Group, Rando Allikmets, Sandro Banfi, Frans P. M. Cremers, Rob W. J. Collin, Elfride De Baere, Hakon Hakonarson, Susanne Kohl, Carlo Rivolta, Dror Sharon, The Associated Clinical Study Group, Maria C. Alonso-Cerezo, Maria J. Ballesta-Martinez, Sergi Beltran, Carmen Benito Lopez, Jaume Català-Mora, Claudio Catalli, Carmen Cotarelo-Perez, Miguel Fernandez-Burriel, Ana Fontalba-Romero, Enrique Galán-Gómez, Maria Garcia-Barcina, Loida M. Garcia-Cruz, Blanca Gener, Belen Gil-Fournier, Nancy Govea, Encarna Guillen-Navarro, Ines Hernando Acero, Cristina Irigoyen, Silvia Izquierdo-Álvarez, Isabel Llano-Rivas, Maria A. López-Ariztegui, Vanesa Lopez-Gonzalez, Fermina Lopez-Grondona, Loreto Martorell, Pilar Mendez-Perez, Maria Moreno-Igoa, Raluca Oancea-Ionescu, Francesc Palau-Martinez, Guiomar Perez de Nanclares, Feliciano J. Ramos-Fuentes, Raquel Rodriguez-Lopez, Montserrat Rodriguez-Pedreira, Lydia Rodriguez-Peña, Berta Rodriguez-Sanchez, Jordi Rosell, Noemi Rosello, Raquel Saez-Villaverde, Alfredo Santana, Irene Valenzuela-Palafoll, Eva Villota-Deleu, Blanca Garcia-Sandoval, Pablo Minguez, Almudena Avila-Fernandez, Marta Corton, Carmen Ayuso

**Affiliations:** 1 Department of Genetics, Health Research Institute-Fundación Jiménez Díaz University Hospital, Universidad Autónoma de Madrid (IIS-FJD, UAM), Madrid, Spain; 2 Center for Biomedical Network Research on Rare Diseases (CIBERER), Instituto de Salud Carlos III, Madrid, Spain; 3 Department of Ophthalmology, Health Research Institute-Fundación Jiménez Díaz University Hospital, Universidad Autónoma de Madrid (IIS-FJD, UAM), Madrid, Spain; 4 Department of Genetics, Health Research Institute-Fundación Jiménez Díaz University Hospital, Universidad Autónoma de Madrid (IIS-FJD, UAM), Madrid, Spain; 5 Center for Biomedical Network Research on Rare Diseases (CIBERER), Instituto de Salud Carlos III, Madrid, Spain; 6 Medical Genetics Unit, Pediatrics Service, Hospital Clínico Universitario Virgen de la Arrixaca, IMIB-Arrixaca, Murcia, Murcia Spain; 7 CNAG-CRG, Center for Genomic Regulation, The Barcelona Institute of Science and Technology, Barcelona, Catalonia Spain; 8 Genetics Section, Hospital Universitario Carlos Haya, Málaga, Andalusia Spain; 9 Department of Genetics, Hospital de la Santa Creu I Sant Pau, Universitat Autònoma de Barcelona, Barcelona, Catalonia Spain; 10 Ophthalmology Service, Hospital Sant Joan de Déu, Barcelona, Catalonia Spain; 11 Department of Medical Genetics, Hospital Universitario Cruces, Bilbao, Basque Country Spain; 12 Clinical Genetics Unit, Hospital Universitario Clínico San Carlos, Madrid, Madrid, Spain; 13 Genetics Unit, Hospital de Mérida, Mérida, Badajoz, Extremadura Spain; 14 Genetics Service, Hospital Universitario Marqués de Valdecilla (HUMV), Santander, Cantabria Spain; 15 Clinical Genetics Unit, Pediatrics Service, Hospital Universitario de Badajoz, Badajoz, Extremadura Spain; 16 Molecular Genetics Unit, Hospital de Terrassa, Consorci Sanitari de Terrassa, Terrassa, Catalonia Spain; 17 Genetics Unit, Hospital Universitario de Basurto, Osakidetza Basque Health Service, Bilbao, Basque Country Spain; 18 Clinical Genetics Unit, Complejo Hospitalario Insular-Materno Infantil, Las Palmas de Gran Canaria, Canary Islands, Spain; 19 Department of Genetics, Hospital Universitario de Getafe, Madrid, Madrid Spain; 20 Genetics Unit, Hospital Son Espases, Palma de Mallorca, Balearic Islands, Spain; 21 Genetics Deparment, Hospital Universitario Central de Asturias, Oviedo, Asturias Spain; 22 Department of Ophthalmology, Hospital Universitario de Donostia, San Sebastián, Basque Country Spain; 23 Division of Neurosciences, Biodonostia Health Research Institute, San Sebastián, Basque Country Spain; 24 Department of Ophthalmology, University of the Basque Country-UPV/EHU, Vizcaya, Basque Country Spain; 25 Clinical Genetics Department, Hospital Universitario Miguel Servet (HUMS), Zaragoza, Aragon Spain; 26 Cellular Therapy Service, Blood and Tissue Bank, Building Dr. Frederic Duran I Jordà, Barcelona, Catalonia Spain; 27 Musculoskeletal Tissue Engineering Group, Vall D’Hebron Research Institute (VHIR), Universitat Autònoma de Barcelona, Barcelona, Catalonia Spain; 28 Department of Medical Genetics, Complejo Hospitalario de Navarra (CHN), Pamplona, Navarre Spain; 29 Navarra Institute for Health Research (IdiSNA), Pamplona, Navarre Spain; 30 Molecular Genetics Unit, Hospital Sant Joan de Deu, Barcelona, Catalonia Spain; 31 Molecular (Epi)Genetic Lab, Bioaraba Health Research Institute, Araba University Hospital, Vitoria-Gasteiz, Alava, Basque Country Spain; 32 Clinical Genetics and Functional Genomics Unit, Pediatrics Service, Hospital Clinico Universitario “Lozano Blesa”, Facultad de Medicina, Universidad de Zaragoza, Zaragoza, Aragon Spain; 33 Department of Clinical Analysis, Hospital Universitario General de Valencia, Valencia, Valencian Community Spain; 34 Genetics Unit, Hospital Materno Infantil Teresa Herrera, Hospital Universitario de A Coruña, A Coruña, Galicia, Spain; 35 Instituto Investigación Illes Balears (IDISBA), Palma de Mallorca, Balearic Islands Spain; 36 Department of Ophthalmology, Hospital de la Santa Creu I Sant Pau, Universitat Autònoma de Barcelona, Barcelona, Catalonia Spain; 37 Department of Genetics, Hospital Universitario Donostia, San Sebastian, Basque Country Spain; 38 Genetics Unit, Hospital Universitari Vall D’Hebron, Barcelona, Catalonia Spain; 39 Instituto Oftalmológico Fernández-Vega, Oviedo, Asturias Spain; 40 Department of Ophthalmology, Columbia University, New York, NY USA; 41 Department of Pathology and Cell Biology, Columbia University, New York, NY USA; 42 Medical Genetics, Department of Precision Medicine, Università degli Studi della Campania ‘Luigi Vanvitelli’, Naples, Italy; 43 Telethon Institute of Genetics and Medicine, Pozzuoli, Italy; 44 Department of Human Genetics, Radboud University Medical Center, Nijmegen, The Netherlands; 45 Department of Human Genetics and Donders Institute for Brain, Cognition and Behaviour, Radboud University Medical Center, Nijmegen, The Netherlands; 46 Center for Medical Genetics Ghent (CMGG), Ghent University and Ghent University Hospital, Ghent, Belgium; 47 Center for Applied Genomics, Children’s Hospital of Philadelphia, Philadelphia, PA USA; 48 Division of Human Genetics, Children’s Hospital of Philadelphia, Philadelphia, PA USA; 49 Department of Pediatrics, Perelman School of Medicine, University of Pennsylvania, Philadelphia, PA USA; 50 Institute for Ophthalmic Research, Center for Ophthalmology, University of Tübingen, Tübingen, Germany; 51 Clinical Research Center, Institute of Molecular and Clinical Ophthalmology Basel (IOB), Basel, Switzerland; 52 Department of Ophthalmology, University Hospital Basel, Basel, Switzerland; 53 Department of Genetics and Genome Biology, University of Leicester, Leicester, UK; 54 Department of Ophthalmology, Hadassah Medical Center, Faculty of Medicine, The Hebrew University of Jerusalem, Jerusalem, Israel; 55 Department of Maternofoetal Medicine, Genetics and Reproduction, Institute of Biomedicine of Seville (IBIS), University Hospital Virgen del Rocío-CSIC-University of Seville, Seville, Spain; 56 Department of Biochemistry, Genetics and Immunology, Facultad de Biología, Universidad de Vigo, Pontevedra, Galicia Spain; 57 Research Group on Rare Diseases and Pediatric Medicine, Instituto de Investigacion Sanitaria Galicia Sur (IISGS), Vigo, Galicia Spain; 58 Centro de Investigaciones Biomédicas (CINBIO), Universidad de Vigo, Vigo, Galicia Spain

**Keywords:** Molecular medicine, Genetics research, Clinical genetics, Hereditary eye disease

## Abstract

Inherited retinal diseases (IRDs), defined by dysfunction or progressive loss of photoreceptors, are disorders characterized by elevated heterogeneity, both at the clinical and genetic levels. Our main goal was to address the genetic landscape of IRD in the largest cohort of Spanish patients reported to date. A retrospective hospital-based cross-sectional study was carried out on 6089 IRD affected individuals (from 4403 unrelated families), referred for genetic testing from all the Spanish autonomous communities. Clinical, demographic and familiar data were collected from each patient, including family pedigree, age of appearance of visual symptoms, presence of any systemic findings and geographical origin. Genetic studies were performed to the 3951 families with available DNA using different molecular techniques. Overall, 53.2% (2100/3951) of the studied families were genetically characterized, and 1549 different likely causative variants in 142 genes were identified. The most common phenotype encountered is retinitis pigmentosa (RP) (55.6% of families, 2447/4403). The most recurrently mutated genes were *PRPH2*, *ABCA4* and *RS1* in autosomal dominant (AD), autosomal recessive (AR) and X-linked (XL) NON-RP cases, respectively; *RHO*, *USH2A* and *RPGR* in AD, AR and XL for non-syndromic RP; and *USH2A* and *MYO7A* in syndromic IRD. Pathogenic variants c.3386G > T (p.Arg1129Leu) in *ABCA4* and c.2276G > T (p.Cys759Phe) in *USH2A* were the most frequent variants identified. Our study provides the general landscape for IRD in Spain, reporting the largest cohort ever presented. Our results have important implications for genetic diagnosis, counselling and new therapeutic strategies to both the Spanish population and other related populations.

## Introduction

Inherited retinal diseases (IRDs) are one of the most heterogeneous clinical and genetical disorders known among all human medical conditions, characterized by the progressive loss of photoreceptor cells, resulting in severe visual impairment^1^. IRDs are classified as rare diseases, and their estimated prevalence is about 1 in 1000^2^–4000^1^. IRDs can be classified according to different clinical or genetic criteria, based upon the primary retinal cell affected (rods, cones, retinal pigment epithelium (RPE), bipolar cells or ganglion cells), the ophthalmological findings and/or the affected gene found after the genetic testing.

All modes of inheritance can be observed (autosomal dominant (AD), autosomal recessive (AR), X-linked (XL), including rare non-Mendelian forms such as mitochondrial or digenic inheritance patterns). Age of onset of first symptoms (from early childhood to adulthood), rate of progression, association with extra-ocular symptoms (non-syndromic versus syndromic forms) or causative gene can also help to subclassify the different phenotypes^3^. The most prevailing form of IRDs is Retinitis Pigmentosa (RP [MIM: 268000]), which is estimated to affect approximately 1.5 million people worldwide^4^. RP begins with the degeneration of rod photoreceptors, resulting in night blindness and characteristic pigmentary changes in the peripheral retina. This is also considered a rod-cone dystrophy due to the subsequent cone photoreceptor death in later stages. Other forms of IRD, including cone-dominated diseases, are characterized by photophobia, reduced visual acuity and impaired colour vision (i.e. cone dystrophy (CD), achromatopsia, blue cone monochromatism), as well as generalized retinal degeneration involving simultaneously both cones and rods such as in rod-cone or cone-rod dystrophies (CRD). The most severe form of non-syndromic IRDs is Leber congenital amaurosis (LCA) characterized by congenital or early childhood blindness. Other IRD forms are characterized by central vision loss affecting primarily the macula and are therefore acknowledged as Macular Dystrophies (MD), such as Stargardt disease (STGD1 [MIM: 248200]) and Best Vitelliform Macular Dystrophy (VMD2 [MIM: 153700])^1^.

Conversely, syndromic IRDs are subclassified according to the type of syndrome. The most prevalent is Usher syndrome (sensorineural hearing loss and RP) which can be further subclassified to Usher type I (USH1 [MIM: 276900]), type II (USH2 [MIM: 276901]) and type III (USH3 [MIM: 276902]), and other syndromes such as Bardet-Biedl (BBS [MIM: 209900]) or Alström (ALMS [MIM: 203800])^3^.

Since the identification of rhodopsin (*RHO* [MIM: 180380]*)* in 1990 as the first gene involved in the development of AD-RP^5^, 270 genes have been additionally described as causative of IRDs (RetNet, Retinal Information Network; https://sph.uth.edu/retnet/; accessed on March 2020). Some of these genes have been reported in only few families worldwide; therefore, their individual contribution to IRD prevalence is relatively small. The most prevalent IRD-causing genes across all populations are *ABCA4* (MIM: 601691), *RHO*, *USH2A* (MIM: 608400) and *RPGR* (MIM: 312610)*,* which account for high percentages of some of the IRD subtypes, i.e. 70–71% in STGD1/AR-MD/AR-CRD^6^, 19–25% of AD-RP, 10% of AR-RP, and 70% of XL-RP, respectively^1^. In addition, although the majority of the causative variants are private, some are more frequent, especially in Spanish families, such as *USH2A* (GenBank: NM_206933.3) c.2299delG (p.Glu767SerfsTer21) and c.2276G > T (p.Cys759Phe)^7^ or *ABCA4* (GenBank: NM_000350.3) c.3386G > T (p.Arg1129Leu) and c.5882G > A (p.Gly1961Glu)^6^.

The aim of this study is to present a comprehensive overview of the largest cohort of IRD patients ever reported worldwide and related to IRD in the Spanish population. The presented data includes the presumed inheritance pattern for the different phenotypic subtypes, mutational spectrum, prevalence of genes carrying likely pathogenic variants and the recurrence of disease related variants.

## Results

### Prevalence of IRD in Spain

**Figure 1:**
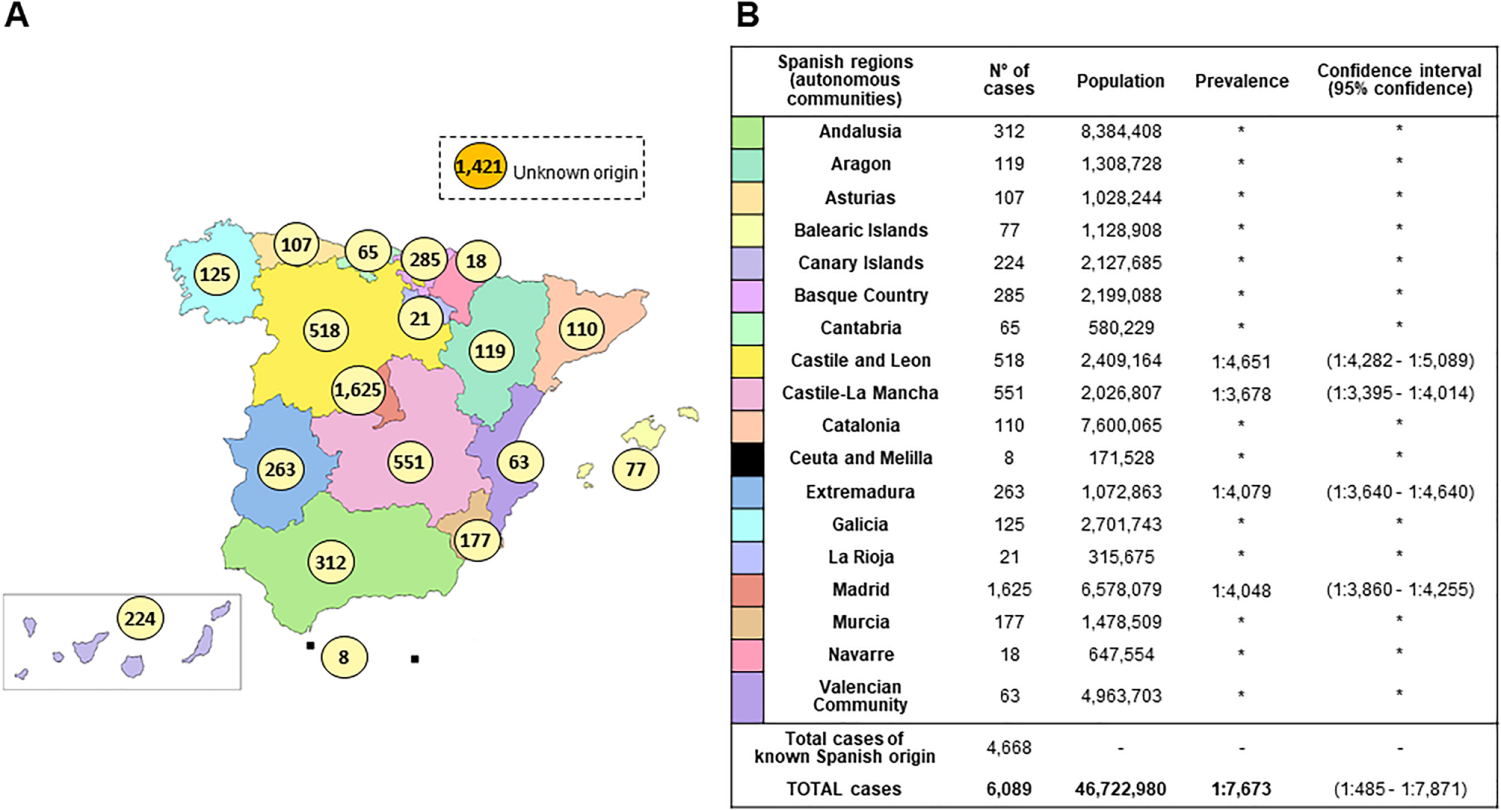
IRD affected cases distribution and estimated prevalence in Spain. (**A**) Distribution of RP/NON-RP affected cases across Spain. Total cases: 6089 (known Spanish origin: 4668; unknown origin: 1421). Spanish map modified using image editor from https://www.veomapas.com/mapa-mudo-de-las-comunidades-autonomas-de-espana-m103.html. (**B**) IRD estimated prevalence in Spanish regions. Data was obtained from the number of cases we had in our Hospital Service and the recorded population in the different regions. *Inconclusive data.

The number of cases diagnosed as having IRD in our hospital (until August 2019) was 6089, and the last Spanish population registry accounted for 46,722,980 habitants giving us a minimal prevalence of 1:7673 (confidence interval (CI):1:7485–1:7871). Regional distribution of cases and prevalence can be seen in Fig. 1A,B. Considering a worldwide IRD prevalence of 1:1000^2^–4000^1^, our cohort would represent 20–53% of the total patients with IRD in Spain as shown in Supplementary Table S1.

For non-syndromic IRD, our cohort was grouped in 2 categories: 1703 affected individuals (1335 families), in cone-dominated phenotypes—hereafter “NON-RP”—and 3561 affected cases from 2447 unrelated families within primarily rod affection—hereafter “RP”. This resulted in a minimal prevalence of 1:27,436 (CI:1:26,192–1:28,804) and 1:13,121 (CI:12,704–13,566) for NON-RP and RP, respectively. Additionally, syndromic IRD forms represented the smallest fraction accounting for 13.6% of the patients with 825 affected individuals (621 families), resulting in a minimal prevalence of 1:56,634 (CI:1:53,016–1:60,781).

In our cohort, we have a higher proportion of cases from Madrid area (26.7%; 1625/6089).

### Initial classification of IRD families by clinical type and suspected mode of inheritance prior to genetic testing

**Figure 2:**
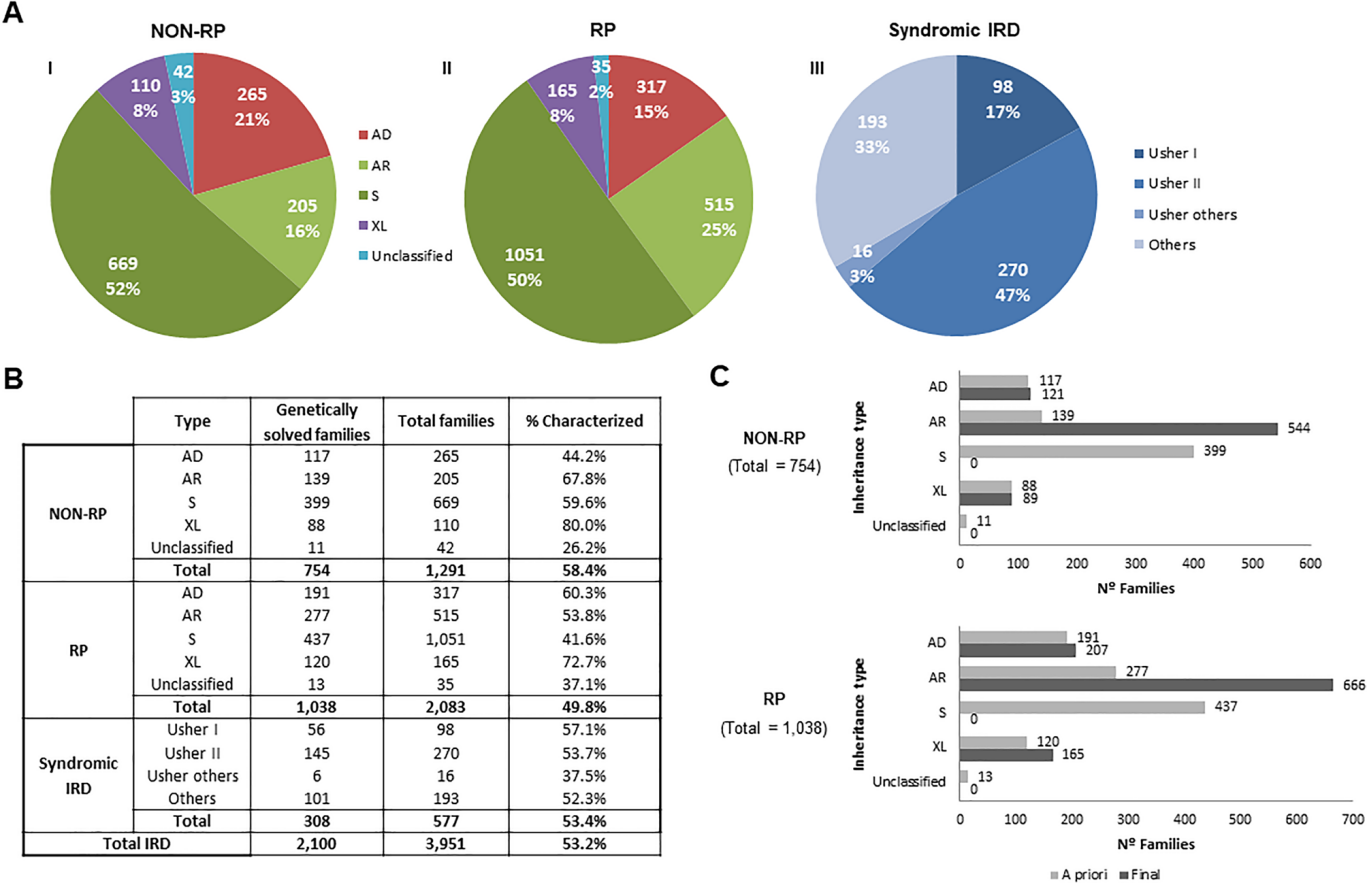
“A priori” and final classification of IRD affected cases. (**A**) “A priori” classification of IRD families with data obtained from the clinical and family history of the patients, before performing molecular tests: NON-RP (I), RP (II), and syndromic IRD (III), and subclassification according to the inheritance type in case of RP and NON-RP, and type of syndrome in case of syndromic IRD. Data obtained with the clinical and familiar history of the patients, before performing molecular tests. *AD* Autosomal Dominant, *AR* autosomal recessive, *S* sporadic, *XL* X-linked. (**B**) Proportion of genetically solved NON-RP, RP, syndromic IRD and total IRD. The diagnostic ratio in the different group of NON-RP and RP by the type of “a priori” inheritance and in the different group of syndromic IRD by type of syndrome is indicated. (**C**) Genetically solved families. Comparison of inheritance classification before (light gray) and after (dark gray) the molecular study was performed.

Non-syndromic NON-RP and RP cases were categorized by the mode of inheritance (Fig. 2A-I and A-II). Syndromic IRD were categorized by the specific type of suspected syndrome (Fig. 2A-III), instead of inheritance type, given that most of them were sporadic (53.6%) or had recessive inheritance (40.2%). The remaining 6.2% corresponded to dominant (0.5%), X-linked (0.7%), mitochondrial inherited disease (0.2%) or non-classificable cases (4.8%).

According to this “a priori” diagnosis based on the clinical and familial history of the patients, the main inheritance pattern in non-syndromic NON-RP and RP was recessive or sporadic, representing the 68% and 75% of cases, respectively. Autosomal dominant and X-linked forms accounted for 21% and 8% for NON-RP and 15% and 8% for RP, respectively (Fig. 2A-I). Families with no familiar data were annotated as unclassified. Non-syndromic RP represents the most common phenotype, representing 55.6% of families in our cohort.

In the present cohort, 47% of the syndromic IRD index cases (270/577) suffered from USH2, followed by 17% USH1 (98/577), as well as other very rare syndromes like some atypical forms of Usher syndrome (3%; 16/577) and ciliopathies such as BBS or ALMS (16%; 90/577). A miscellanea of non-ciliopathic syndromes or unclassified symptoms were presented in 103 index cases.

### Molecular studies

#### Diagnostic yield

Genetic testing was performed in a total of 3951 index cases with available DNA^6–11^ (89.7% of the total cohort), including 1291 NON-RP, 2083 RP, and 577 syndromic IRD patients as shown in Supplementary Fig. S1. The genetic analysis procedures evolved over time since novel genetic approaches were implemented in our laboratory. A definite genetic diagnosis was established for 53.2% (2100/3951) of cases. For NON-RP families, 754 out of 1291 (58.4%) obtained a genetic diagnosis; in RP the molecular cause was identified in 1038 out of 2083 (49.8%). Similarly, 53.4% (308/577) of syndromic IRD families were genetically solved (Fig. 2B).

### Final inheritance pattern and reclassification of characterized families

The identification of the causative gene allowed us to reclassify the inheritance mode in 8.2% (146/1792) of the NON-RP and RP families, thus establishing the final inheritance type (Supplementary Table S2). A comparison between the “a priori*”* suspected inheritance based on the pedigree and the final inheritance suggested by the molecular diagnosis was performed in characterized NON-RP and RP families (Fig. 2C). As expected, most sporadic NON-RP (n = 378) and RP cases (n = 379) were confirmed as having AR inheritance after the genetic testing. The rest of the S cases were reclassified to AD (n = 43) and XL (n = 36) (Supplementary Table S2). Twenty-four cases (11 NON-RP and 13 RP) with an initial unknown mode of inheritance were classified as: AD (n = 5), AR (n = 16), and XL (n = 3) after the molecular testing.

#### Gene landscape

**Figure 3:**
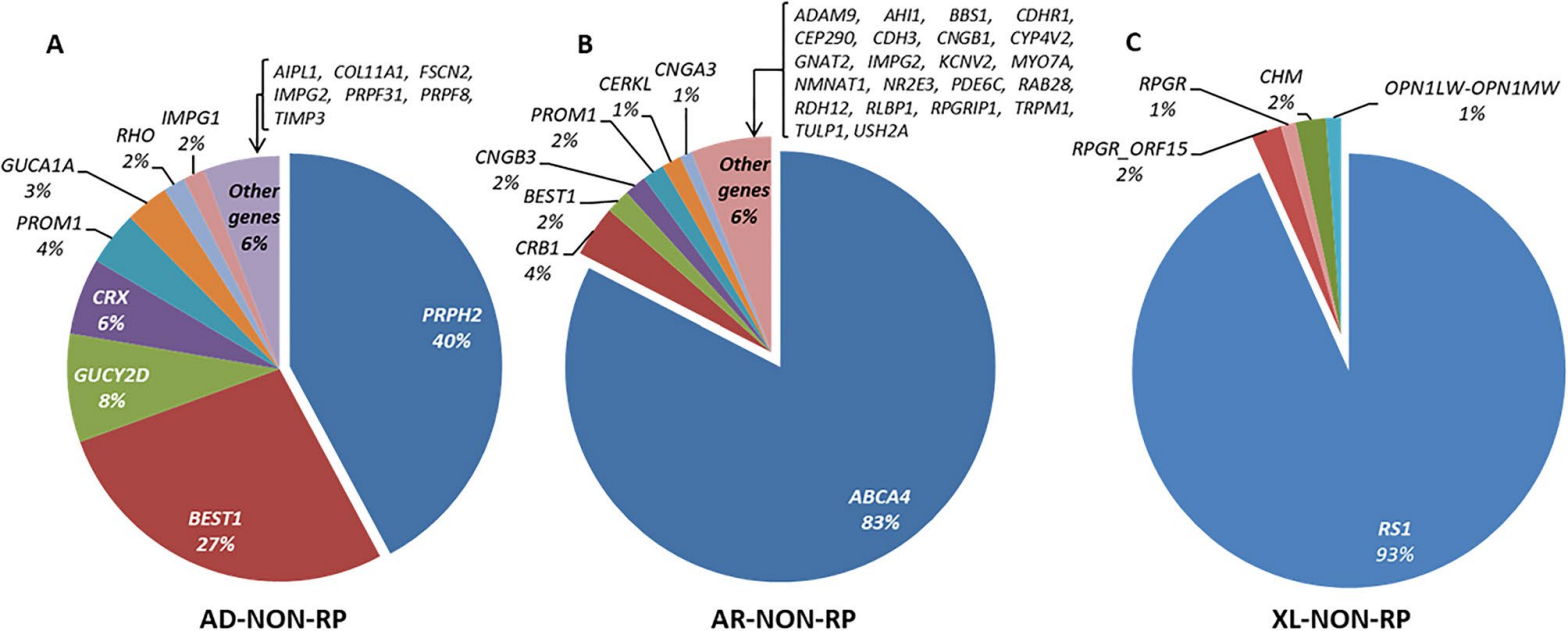
Classification of mutated genes in the genetically solved NON-RP affected cases. Below each gene is given the percentage that each gene was mutated in the cohort. Total characterized families: AD-NON-RP: 121; AR-NON-RP: 544; XL-NON-RP: 89.

**Figure 4:**
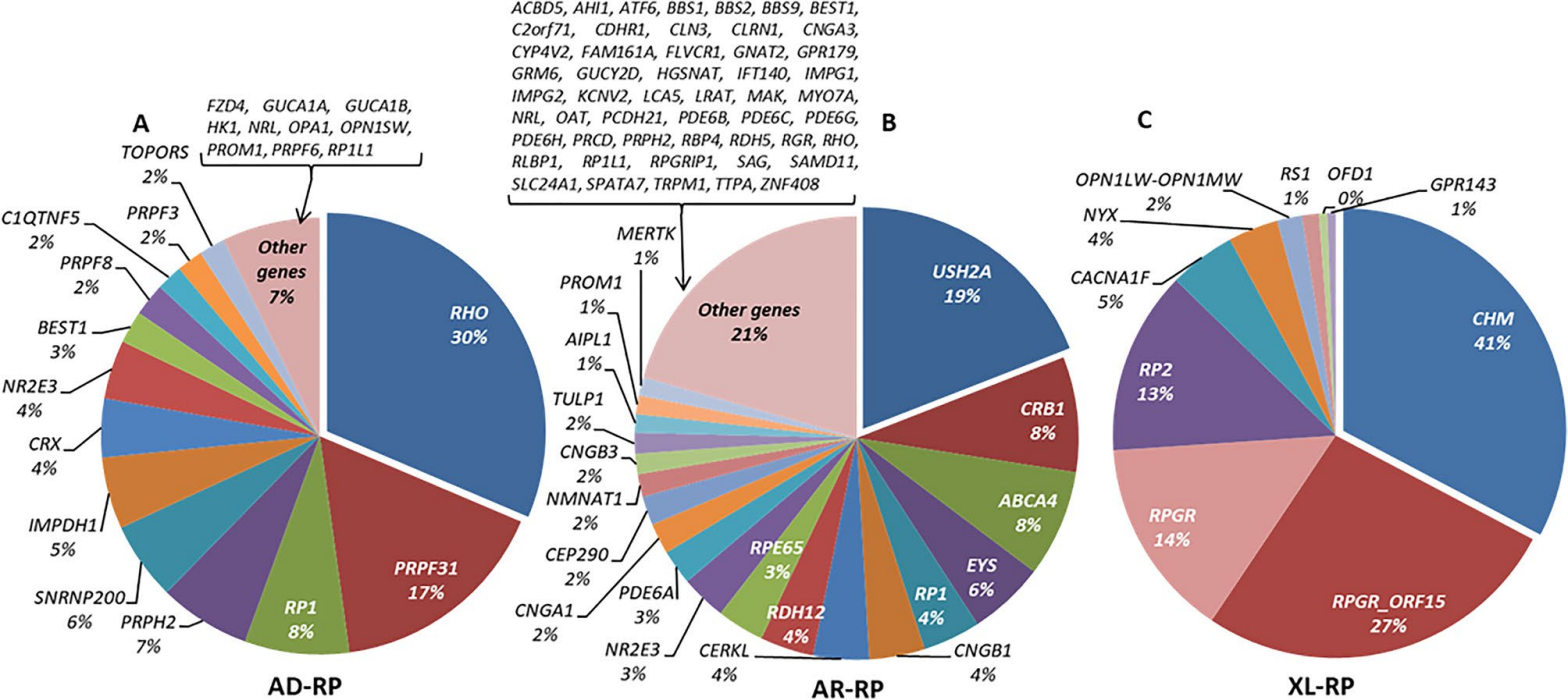
Classification of mutated genes in the genetically solved RP affected cases. Below each gene is given the percentage that each gene was mutated in the cohort. Total characterized families: AD-RP: 207; AR-RP: 666; XL-RP: 165.

**Figure 5:**
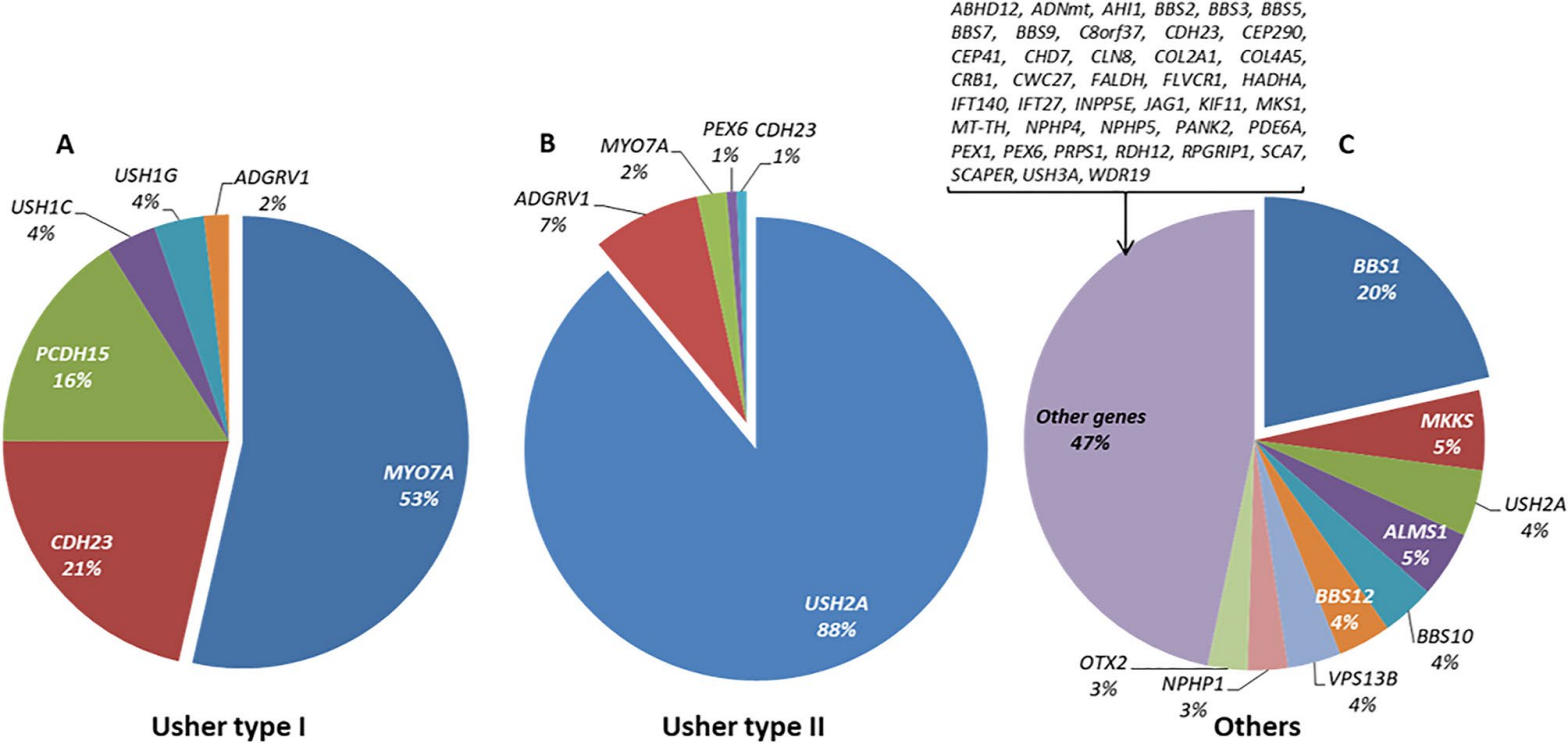
Classification of mutated genes in the genetically solved syndromic IRD affected cases. Below each gene is given the percentage that each gene was mutated in the cohort. Total characterized families: Usher I: 56; Usher II: 145; Others (including atypical Usher): 107.

In total, 1549 different pathogenic and likely pathogenic variants were identified in 142 different genes. These included SNVs (Single Nucleotide Variants) and CNVs (Copy Number Variants). As showed in Figs. 3, 4, 5, there was a wide spectrum of genes implicated in IRD, 121 of them represented in 1% or less of the cohort. The 5 most frequent mutated genes were *ABCA4, USH2A, RS1* (MIM: 300839)*, CRB1* (MIM: 604210) and *RHO.*

In NON-RP families, *PRPH2* (MIM: 179605), *ABCA4* and *RS1* were the most commonly mutated genes, explaining 42.2%, 82.7% and 93.3% of the AD, AR and XL forms, respectively as shown in Supplementary Table S3.

For non-syndromic RP, 207 AD-RP families were genetically characterized with heterozygous variants identified in one of 23 genes found. The most frequent mutated genes were *RHO* (65/207; 31.4%) and *PRPF31* (MIM: 606419) (34/207; 16.4%). In AR-RP families, the most common gene mutated was *USH2A* (127/666; 19.1%). However, the number of other disease genes detected was very high (N = 70) in the 666 families characterized. For XL-RP families, both the number of cases and variety of genes were low (N = 9) as shown in Supplementary Table S4. For these patients, 41.2% (68/165) of the index cases (all males) carried a hemizygous pathogenic variant in *RPGR* (44 *RPGR_ORF15* and 24 in the rest of *RPGR* regions)*,* and 32.7% (54/165) in *CHM* (MIM: 300390).

A total of 53 cases out of the total 2100 (2.5%) were clinically reconsidered and reclassified after genetic testing: 12 NON-RP were reclassified as RP (9/754; 1.2%; characterized with *RHO, FSCN2, PRPF8, AHI1, CNGB1, TRPM1* and *CHM*) or as syndromic IRD (3/754; 0.4%; *COL11A1, CDH3* and *MYO7A*) and 37 RP as NON-RP (32/1038; 3.1%; *C1QTNF5, GUCA1A, CNGB3, CNGA3, ACBD5, GNAT2, PDE6C, ATF6, PDE6H, RS1* and *OPN1LW-OPN1MW*), syndromic IRD (1/1038; 0.1%; *MYO7A*) and other visual diseases, such as exudative familiar vitreoretinopathy, optic atrophy, albinism and gyrate atrophy (4/1038; 0.4%; *FZD4, OPA1, GPR143* and *OAT*). Moreover, 4 syndromic cases were reclassified to RP group (4/308; 1.3%; *RDH12, PDE6A* and *RPGRIP1*).

Among syndromic IRD families, the causative gene was identified in 56 families with a diagnosis of USH1, 145 of USH2, 6 of atypical Usher, and 101 of other syndromes including Bardet-Biedl or Alström syndrome, respectively. In USH1, biallelic variants in *MYO7A* (MIM: 276903) were identified in 30 out of 56 patients (53.5%). *USH2A* defects were the main cause of USH2 in 90% (129/145) of the patients. The group “others” was a clinically heterogeneous group of non-Usher cases with a total of 48 involved genes, with *BBS1* (MIM: 209901) being the most frequent one (N = 23), as shown in Supplementary Table S5.

In addition to clinical and genetic heterogeneity, the group of “others” included in syndromic IRD also presented unusual modes of inheritance, such as triallelism. In our cohort, 4 possible triallelic cases have been identified, all of them diagnosed with BBS, 3 patients carrying biallelic *BBS1* variants together with one allele in *MKKS* (MIM: 604896)^9,12^ and one additional case carrying biallelic *MKKS* variants and one allele in *BBS5* (MIM: 603650). The phenotypic modifier effect of the triallelism could only be stablished in two of the 4 families. One has two affected siblings with different phenotypic severity that correlates with the presence of the third allele; and in the other both affected showed triallelism and have the same clinical manifestation. Although, it could not be stablished in the rest of the families, as they were sporadic cases.

#### Most frequent variants

Our findings reflect the high allelic heterogeneity in IRD. We identified 458 different disease-causing variants in 45 genes in cases “a priori” classified as NON-RP, as well as 836 in 94 genes in the “a priori” RP cases and 295 in 55 genes in the “a priori” syndromic IRD. The most common pathogenic variant detected in our NON-RP cohort was the previously known missense change *ABCA4* c.3386G > T (p.Arg1129Leu) (180 mutated alleles of 3,618; 5% of the total pathogenic alleles), presented in 21.5% (162/754) of the characterized families, in homozygous or compound heterozygous state in 18 and 144 families, respectively (Supplementary Table S6). Among the RP families, the most prevalent pathogenic variant was the missense change *USH2A* c.2276G > T (p.Cys759Phe), identified in 106 alleles in 8.4% of the solved families (87/1038); in 19 cases in homozygous and in 68 cases in compound heterozygous state. In addition, there were 15 other variants present in more than 10 genes (Supplementary Table S6). Some of the mutated genes overlap in NON-RP (Fig. 3), RP (Fig. 4) and syndromic IRD (Fig. 5).

#### Disease-causing variant distribution in Spain

Analysis of causing variants by the different Spanish regions resulted in a wide variety of disease-causing variants. Table 1 shows variants detected in more than 5% of characterized families, by Spanish regions. All these variants are depicted in the Supplementary Table S6 of NON-RP and RP most frequent causing variants, except for the nonsense variant *PRCD* (OMIM: 610598) (GenBank: NM_001077620.3) c.64C > T (p.Arg22Ter)*,* that was found in homozygosity in 3 families, representing 7.2% (6/83) of the identified alleles from Murcia.

**Table 1:** Variants detected in > 5% of NON-RP, RP and syndromic IRD by Spanish regions.

	Spanish region	Gene	Nucleotide change	Amino acid change	Nº of alleles	Nº of total alleles of this gene found in the region	Frequency in the region
NON-RP	Basque Country	*ABCA4*	c.3386G > T	p.Arg1129Leu	18	93	19.40%
		*ABCA4*	c.5882G > A	p.Gly1961Glu	5		5.40%
	Castile-La Mancha	*ABCA4*	c.3386G > T	p.Arg1129Leu	8	60	13.30%
	Madrid	*ABCA4*	c.3386G > T	p.Arg1129Leu	47	373	12.60%
	Murcia	*ABCA4*	c.3386G > T	p.Arg1129Leu	8	71	11.30%
		*ABCA4*	c.5882G > A	p.Gly1961Glu	7		9.90%
Non-syndromic RP and syndromic IRD	Murcia	*USH2A*	c.2276G > T	p.Cys759Phe	11	83	13.30%
		***PRCD***	c.64C > T	p.Arg22Ter	6		7.20%
	Canary Islands	*NR2E3*	c.932G > A	p.Arg311Gln	8	83	9.60%
	Castile-La Mancha	*USH2A*	c.2276G > T	p.Cys759Phe	18	202	8.90%
	Madrid	*USH2A*	c.2276G > T	p.Cys759Phe	50	714	7.00%
	Extremadura	*USH2A*	c.2276G > T	p.Cys759Phe	5	87	5.70%

Regions with variants detected below this percentage are not represented in the table, and neither are regions with less than 50 mutated alleles in total or variants that were presented less than 5 times. In bold the variant which not appeared as one of the most frequent variants in Supplementary Table S6.

## Discussion

This is the first and largest comprehensive study addressing the prevalence and epidemiology of IRD in the Spanish population. The cohort here described, comprising 6089 cases from 4403 unrelated families, is not based on a national registry of IRD patients, but it is the outcome of a very wide recruiting effort of a single center over the last 28 years. An increasing number of centers are currently performing clinical and/or genetic diagnosis of IRD in Spain, therefore our cohort did not reflect all of IRD patients in our country. Hence, to date, no accurate data about the IRD prevalence in the Spanish population is available.

In terms of representation of patients from the different Spanish regions, our cohort reflects a biased recruitment, being enriched with patients from Madrid and the surrounding regions (i.e. Castile and Leon, Castile-La Mancha, and Extremadura) probably due to the fact that our hospital has been their referral center during most of the time of the study. Other areas like Andalusia, Catalonia, Navarre or the Valencian Community had different referral centers and genetic testing is performed locally. In spite of these limitations, the large sample size of our cohort and the exhaustive molecular analysis performed over the years, together with an overall low genetic heterogeneity in the Spanish population^13^, have allowed a straightforward extrapolation of prevalent genes and/or variants in IRD. Considering a worldwide prevalence of 1:1000^2^–4000^1^ and an estimated Spanish population of 46.7 million, our cohort would represent 20–53% of the total patients with IRD in Spain. Despite numerous studies about the characteristics of the different IRDs in Spain, such as NON-RP and RP have been partially published, still no global overview of NON-RP and RP diseases using a representative cohort has been addressed yet before in our country.

Several studies on IRD have been performed globally (Supplementary Table S7) and, in the two last years, some including big cohorts^14–16^ or meta-analysis^17^ have been published, reporting more than 125 genes explaining 55–62% of the families using several molecular techniques to achieve that^14–16^ as it could be also seen in this study.

Other studies focused on stablishing the prevalence of IRD in certain regions has been performed in Western countries and in cohorts of non-syndromic RP, including Western Australia (1:6000)^18^ or Maine (1:4756)^19^, as well as in cohorts with general IRD and an estimated prevalence of 1:3454 in Denmark^20^ or 1:3856 in Norway^21^. However, this prevalence has been reported in areas and populations with low rates of consanguinity and could be higher when consanguinity rate increases^2^, which is not the common scenario in Spain nowadays.

Our study identified AR inheritance as the most common mode of inheritance for non-syndromic IRD, explaining up to 70–75% NON-RP and RP subcohorts (Fig. 2A-I and A-II). By contrast, only 7% of our NON-RP and RP families are explained by X-linked genes. These results were consistent with previous studies published^18–22^. Besides, some cases could be explained by different molecular mechanisms as the pseudodominance, incomplete penetrance or the presence of two variants in an AR gene in AD a priori families^8^, so extended segregation analysis within these families are needed. Additional non-Mendelian transmission patterns were only found in exceptionally rare cases with syndromic IRD, including 3 families carrying variants affecting the mitochondrial DNA and 4 cases with apparent triallelism in BBS-associated genes. Within syndromic IRD group, most of the cases were explained by AR biallelic monogenic inheritance. Similar to previous published studies from other countries^3,23^, Usher syndrome was the most prevalent form of syndromic IRD in our cohort, and more specifically, USH2, representing almost half of the total syndromic IRD families.

The overall diagnostic rate of 53.2% obtained here is similar to other studies previously reported (50–70%)^24–26^. Molecular studies allowed the identification of the genes responsible for the disease and the reclassification of the inheritance type. In our work, 8.2% of the patients were reclassified after the detection of the disease-causing variants in genes with a specific inheritance pattern. All of those were or have been previously validated. Moreover, in all the characterized sporadic cases a more accurate genetic classification and counselling could be done^8^. Additionally, a 2.5% were clinically reclassified after the genetic testing, due to a poor clinical data acquisition at the origin center. So, identification of the genetic cause of the disease represents a hallmark for the patients, firstly, regarding genetic counselling and the risk of affectation for other relatives; and secondly, given the possibility of future recruitments for clinical trials targeting specific genes and variants.

A total of 142 different genes were identified as the cause of IRD in our study, but it is important to notice that each subgroup of the cohort (AD, AR and XL NON-RP and RP) has an enrichment of characterized cases in specific genes.

For instance, *PRPH2* was mutated in more than a third of AD-NON-RP families, followed by *BEST1* (MIM: 607854). As expected, *ABCA4* was the most prevalent gene in AR-NON-RP families. Recent studies in Norway^21^ and Korea^22^ also identified this gene as one of the most prevalent mutated genes. A study published by Birtel et al.^27^ in patients with MD and cone/cone-rod dystrophy showed a similar distribution of mutated genes, with *ABCA4*, *PRPH2* and *BEST1* responsible for 74% of their solved cases. For the XL-NON-RP subcohort, *RS1* was the most frequently mutated gene.

Non-syndromic RP presented a wider spectrum of causative genes, with *RHO*, *USH2A* and *RPGR* (*RPGR_ORF15* and the rest of *RPGR* regions) being the most prevalent ones in AD-RP, AR-RP and XL-RP subcohorts, respectively. Our findings are in line with those published in other studies^8,28^. For instance, Hartong et al.^3^, showed as well *MYO7A*, *USH2A* and *BBS1* to be the most frequently mutated genes in USH1, USH2 and BBS, respectively. Other studies in different populations highlighted different genes as the most representative in their IRD cohorts. For example, Eisenberger et al.^24^ described *RP1* (MIM: 603937) (11.3%) and *EYS* (MIM: 612424) (9.4%) as the most frequent genes in German patients with AR-RP, and Kim et al.^22^ detected that *EYS* (22%) and *PDE6B* (MIM: 180072) (17%) are most frequently involved in AR-RP in Korean patients. *EYS* was also the most prevalent causative gene in the Japanese population studied by Maeda et al.^29^, implicated in 21 out of 33 AR-RP patients (63.6%), whereas in our population *EYS* was mutated in 5.5% of the families with “a priori” AR-RP diagnosis, being the fourth most frequent gene, after *USH2A*, *CRB1* and *ABCA4*. However, the order of the causative genes in AR-RP changes after reviewing the clinical data of *ABCA4* related IRD patients, since they were mostly reclassified as NON-RP, downgrading *EYS* as the third most common gene in AR-RP in our population. This result supports an eastward gradient in the frequency of *EYS* variants in patients throughout the world and within Europe, being more frequent in Germany than in Spain.

The most frequent causing variants detected in our study appeared, as expected, in *ABCA4* and *USH2A*, the most prevalent mutated genes in the Spanish population^6–8,30^. *ABCA4* c.3386G > T (p.Arg1129Leu) is a variant almost exclusively found in Spanish NON-RP patients^6,30^, being probably a Spanish founder mutation^31,32^. However, *USH2A* c.2276G > T (p.Cys759Phe) is not exclusive from the Spanish population and has been reported in other populations^33^.

According to the geographical distribution of the variants within the country, no differences between regions were observed. In NON-RP, the two most common *ABCA4* variants were also the most represented in regions with variant frequencies above 5%. Meanwhile, in RP we found a higher representation of the most common *USH2A* variant, which appeared above 5% of the total alleles in four regions. Finally, two variants appeared to be more frequent in some regions, i.e. *PRCD* c.64C > T (p.Arg22Ter) in Murcia and *NR2E3* (MIM: 604485) (GenBank: NM_014249.4) c.932G > A (p.Arg311Gln) in the Canary Islands, where a founder effect could be happening.

Our results delineate the genetic background of the Spanish IRD patients, indicating a wide range of causative genes involved in the disease. Some of the causing variants identified are also frequent in Europe. Some examples include the *ABCA4* c.5882G > A (p.Gly1961Glu), reported with high prevalence in the Italian, German and Spanish populations^30,34,35^; the c.2276G > T (p.Cys759Phe) as one of the most frequent variants in *USH2A*, especially in European countries^36^, *USH2A* c.2299delG (p.Glu767SerfsTer21), which is possibly an ancestral European pathogenic variant^37^; *BBS1* (GenBank: NM_024649.5) c.1169T > G (p.Met390Arg), identified previously by Mykytyn et al.^38^ in 22 North American BBS probands with North European ancestry; and *CRB1* (GenBank: NM_201253.3) c.2843G > A (p.Cys948Tyr), identified repeatedly in different European countries^39,40^. Finally, *RHO* (GenBank: NM_000539.3) c.1040C > T (p.Pro347Leu), described in the Italian and French populations^41,42^ and also in non-European cohorts^5,43,44^.

Variants found in individuals from East Mediterranean and Middle-Eastern countries also appeared in our Spanish cohort: *PRCD* c.64C > T (p.Arg22Ter) found by Sharon et al.^14^ in homozygosis in 15 Israeli Muslim Arab families, and by Beheshtian et al.^45^ in a Persian family; and *FAM161A* (MIM: 613596) (GenBank: NM_001201543.2) c.1355_1356delCA (p.Thr452SerfsTer3), which was identified in Jewish families mainly originating from North African countries^46^. As mentioned above, the variant in *PRCD* was found with a higher frequency in the region of Murcia, and this could be due to the settlement of Muslim populations during several centuries during the Middle Ages^13^. *FAM161A* does not have a significant specific geographical distribution in Spain.

Remarkably, we identified three pathogenic variants with high frequency in Spain: *ABCA4* c.3386G > T (p.Arg1129Leu), previously mentioned; *CERKL* (MIM: 608381) (GenBank: NM_201548.5) c.847C > T (p.Leu283Phe), first described by Tuson et al.^47^, and *RP1* c.1625C > G (p.Ser542Ter) previously described originally as a Spanish founder pathogenic variant. These three variants had been scarcely reported outside the Spanish population. In the case of *RP1* c.1625C > G (p.Ser542Ter) variant^48^, because of its presence in 11 out of 244 unrelated families, we can extrapolate that it may very well account for approximately 4.5% of all AR-RP cases in the Spanish population. Other groups also identified this variant in Swiss patients^26^.

In conclusion, this study shows the general landscape of the genetic underpinnings of IRD in Spain and will help design clinical and preventive healthcare approaches to this disorder in our country.

## Materials and methods

### Cohort description

A retrospective analysis was performed including all IRD patients from our Spanish registry at the Fundación Jiménez Díaz University Hospital (FJD, Madrid, Spain) from 1991 until August 2019. This patient registry includes: all patients referred to the Genetic Service at the FJD for genetic diagnostic testing and/or counselling due to a previous clinical suspicion of IRD, and patients without genetic analysis in our unit but identified in the shared electronic clinical history of our same-company hospitals using ICD (International Classification of Diseases) terms. The complete cohort contains 6089 IRD affected cases (including index cases and affected relatives) belonging to 4403 unrelated families as shown in Supplementary Fig. S1.

This study was approved by the Ethics Committee of the FJD under approval number 134/2016_FJD and fulfilled all the tenets of the Declaration of Helsinki and its further reviews. A written informed consent form was obtained from all the patients or their legal guardians.

### IRD classification and diagnostic criteria

During this study, different clinical, demographic and familiar data were collected, including (i) family pedigree; (ii) age at onset of visual acuity loss, extent of visual field loss, night blindness and/or other early symptoms of retinal dystrophy; (iii) presence of any systemic findings suggestive of syndromic forms of IRD; (iv) geographical origin of the patients.

Clinical diagnosis was based on ophthalmic examination, including measurement of best-corrected visual acuity, visual field testing, fundus examination and, if possible, full-field electroretinography, fundus autofluorescence and spectral domain optical coherence tomography scan. NON-RP and RP include non-syndromic IRD, and their clinical classification was done according to previously described criteria^6,8^. NON-RP group include most patients with CD, CRD and achromatopsia, although some of them were included in the RP group due to incomplete phenotyping at the moment of the diagnosis. Non-syndromic LCA cases were also included in RP.

For NON-RP and RP families, an “a priori” inheritance pattern (AD/AR/XL/sporadic (S)) was established according to previously described criteria^1^. The subgroup of XL-RP also included choroideremia cases.

For cases not extensively described in the first referral, a generic classification was made as NON-RP or RP.

Criteria for syndromic IRD diagnosis were previously described^7,9^.

Information about the geographic origin of all the IRD cases from Spain was available in 4668. They are distributed throughout the 17 different Spanish communities (Fig. 1A).

### Inheritance reclassification of IRD cases

After molecular diagnosis, inherited patterns were reviewed and compared with “a priori” data of each family. Statistical analysis between these data sets to assess the global association in the NON-RP group was made using the Fisher’s exact test with a p equal to 0.497. Whereas for the RP group, Chi-square test was used with a p below 0.001. Comparisons for each type of inheritance have also been made with the Fisher’s exact test in the NON-RP Unclassified subgroup and with the Chi-square test in the rest. Fisher’s exact test was used in those cases in which more than 20% of expected values were below than 5, or at least one of the expected frequencies was below 1. Regarding the significance levels chosen, we have a global comparison for which the significance level is the usual threshold of 0.05 and p-value is not corrected, and several post-hoc comparisons for which Bonferroni’s multiple comparisons adjustment is applied, multiplying the p-values by the number of comparisons.

### Prevalence of IRD

In this study, we performed a retrospective analysis of the largest cohort of patients with IRD from Spain, whom were recruited during a period of 28 years by a single center, the FJD. The FJD is a center of reference for molecular diagnosis of IRD from all over the country, especially in some specific autonomous communities, like Castile and Leon, Castile-La Mancha, Extremadura or Madrid. On the other hand, as we take into account other Spanish regions, there are other centers of reference and the prevalence data obtained is inconclusive.

Prevalence was calculated for each clinical type of the disease by dividing the total number of diagnosed IRD cases by the total population in Spain, published by the Spanish Statistical Office (INE; http://www.ine.es) in January 2019.

### Genomic screening

Genomic DNA samples were obtained from the FJD Biobank from a total of 3951 families (89.7%), including 1291 NON-RP, 2083 non-syndromic RP and 577 syndromic IRD families. Molecular studies were performed using different molecular techniques as shown in Supplementary Table S8. According to the technology available and the knowledge on the genetic determinants of IRD at the time of the diagnosis, a maximum of 291 different genes involved in IRD were processed for the molecular characterization (Supplementary S1 Appendix). In these studies, index cases were initially screened, analysed following the American College of Medical Genetics and Genomics (ACMG; https://www.acmg.net/docs/standards_guidelines_for_the_interpretation_of_sequence_variants.pdf) variants classification guidelines. If potentially disease-causing variants were found, segregation analysis was performed when DNA samples from relatives were available.

In the general description of mutated genes and frequent pathogenic variants, only fully molecularly characterized index cases were considered. Patients with a heterozygote allele in a recessive gene were counted as uncharacterized.

The frequency of recurrent IRD causing variants was established considering not only the total Spanish population, but also the different geographical regions of Spain (Fig. 1A), in order to assess the possibility of identifying any endemic or founder effects. Pathogenic variants with a prevalence above 5% in a particular region were recorded, and only those with a higher prevalence were considered for further analysis.

## Supplementary Information

Supplementary information.

## Data Availability

Part of the NGS data are available in public, open access repositories such as the European Genome-Phenome Archive (EGA; https://www.ebi.ac.uk/ega/home; EGAD00001005746 and EGAD00001005498), RD-Connect (https://rd-connect.eu/) and the Collaborative Spanish Variant Server (CSVS; http://csvs.babelomics.org/) as aggregated data. The rest of the data are available upon reasonable request.
