# Author Correction: Genetic landscape of 6089 inherited retinal dystrophies affected cases in Spain and their therapeutic and extended epidemiological implications

**DOI:** 10.1038/s41598-021-89275-4

**Published:** 2021-05-10

**Authors:** Irene Perea-Romero, Gema Gordo, Ionut F. Iancu, Marta Del Pozo-Valero, Berta Almoguera, Fiona Blanco-Kelly, Ester Carreño, Belen Jimenez-Rolando, Rosario Lopez-Rodriguez, Isabel Lorda-Sanchez, Inmaculada Martin-Merida, Lucia Pérez de Ayala, Rosa Riveiro-Alvarez, Elvira Rodriguez-Pinilla, Saoud Tahsin-Swafiri, Maria J. Trujillo-Tiebas, The ESRETNET Study Group, Ana Bustamante-Aragones, Rocio Cardero-Merlo, Ruth Fernandez-Sanchez, Jesus Gallego-Merlo, Ines Garcia-Vara, Ascension Gimenez-Pardo, Laura Horcajada-Burgos, Fernando Infantes-Barbero, Esther Lantero, Miguel A. Lopez-Martinez, Andrea Martinez-Ramas, Lorena Ondo, Marta Rodriguez de Alba, Carolina Sanchez-Jimeno, Camilo Velez-Monsalve, Cristina Villaverde, Olga Zurita, Domingo Aguilera-Garcia, Jana Aguirre-Lamban, Ana Arteche, Diego Cantalapiedra, Patricia Fernandez-San Jose, Liliana Galbis-Martinez, Maria Garcia-Hoyos, Carlos Lombardia, Maria I. Lopez-Molina, Raquel Perez-Carro, Luciana R. J. Da Silva, Carmen Ramos, Rocio Sanchez-Alcudia, Iker Sanchez-Navarro, Sorina D. Tatu, Elena Vallespin, Elena Aller, Sara Bernal, Maria J. Gamundi, Gema Garcia-Garcia, Inmaculada Hernan, Teresa Jaijo, Guillermo Antiñolo, Montserrat Baiget, Miguel Carballo, Jose M. Millan, Diana Valverde, The ERDC Study Group, Rando Allikmets, Sandro Banfi, Frans P. M. Cremers, Rob W. J. Collin, Elfride De Baere, Hakon Hakonarson, Susanne Kohl, Carlo Rivolta, Dror Sharon, The Associated Clinical Study Group, Maria C. Alonso-Cerezo, Maria J. Ballesta-Martinez, Sergi Beltran, Carmen Benito Lopez, Jaume Català-Mora, Claudio Catalli, Carmen Cotarelo-Perez, Miguel Fernandez-Burriel, Ana Fontalba-Romero, Enrique Galán-Gómez, Maria Garcia-Barcina, Loida M. Garcia-Cruz, Blanca Gener, Belen Gil-Fournier, Nancy Govea, Encarna Guillen-Navarro, Ines Hernando Acero, Cristina Irigoyen, Silvia Izquierdo-Álvarez, Isabel Llano-Rivas, Maria A. López-Ariztegui, Vanesa Lopez-Gonzalez, Fermina Lopez-Grondona, Loreto Martorell, Pilar Mendez-Perez, Maria Moreno-Igoa, Raluca Oancea-Ionescu, Francesc Palau-Martinez, Guiomar Perez de Nanclares, Feliciano J. Ramos-Fuentes, Raquel Rodriguez-Lopez, Montserrat Rodriguez-Pedreira, Lydia Rodriguez-Peña, Berta Rodriguez-Sanchez, Jordi Rosell, Noemi Rosello, Raquel Saez-Villaverde, Alfredo Santana, Irene Valenzuela-Palafoll, Eva Villota-Deleu, Blanca Garcia-Sandoval, Pablo Minguez, Almudena Avila-Fernandez, Marta Corton, Carmen Ayuso

**Affiliations:** 1 Department of Genetics, Health Research Institute-Fundación Jiménez Díaz University Hospital, Universidad Autónoma de Madrid (IIS-FJD, UAM), Madrid, Spain; 2 Center for Biomedical Network Research on Rare Diseases (CIBERER), Instituto de Salud Carlos III, Madrid, Spain; 3 Department of Ophthalmology, Health Research Institute-Fundación Jiménez Díaz University Hospital, Universidad Autónoma de Madrid (IIS-FJD, UAM), Madrid, Spain; 4 Department of Genetics, Health Research Institute-Fundación Jiménez Díaz University Hospital, Universidad Autónoma de Madrid (IIS-FJD, UAM), Madrid, Spain; 5 Center for Biomedical Network Research on Rare Diseases (CIBERER), Instituto de Salud Carlos III, Madrid, Spain; 6 Medical Genetics Unit, Pediatrics Service, Hospital Clínico Universitario Virgen de la Arrixaca, IMIB-Arrixaca, Murcia, Murcia Spain; 7 CNAG-CRG, Center for Genomic Regulation, The Barcelona Institute of Science and Technology, Barcelona, Catalonia Spain; 8 Genetics Section, Hospital Universitario Carlos Haya, Málaga, Andalusia Spain; 9 Department of Genetics, Hospital de la Santa Creu I Sant Pau, Universitat Autònoma de Barcelona, Barcelona, Catalonia Spain; 10 Ophthalmology Service, Hospital Sant Joan de Déu, Barcelona, Catalonia Spain; 11 Department of Medical Genetics, Hospital Universitario Cruces, Bilbao, Basque Country Spain; 12 Clinical Genetics Unit, Hospital Universitario Clínico San Carlos, Madrid, Madrid, Spain; 13 Genetics Unit, Hospital de Mérida, Mérida, Badajoz, Extremadura Spain; 14 Genetics Service, Hospital Universitario Marqués de Valdecilla (HUMV), Santander, Cantabria Spain; 15 Clinical Genetics Unit, Pediatrics Service, Hospital Universitario de Badajoz, Badajoz, Extremadura Spain; 16 Molecular Genetics Unit, Hospital de Terrassa, Consorci Sanitari de Terrassa, Terrassa, Catalonia Spain; 17 Genetics Unit, Hospital Universitario de Basurto, Osakidetza Basque Health Service, Bilbao, Basque Country Spain; 18 Clinical Genetics Unit, Complejo Hospitalario Insular-Materno Infantil, Las Palmas de Gran Canaria, Canary Islands, Spain; 19 Department of Genetics, Hospital Universitario de Getafe, Madrid, Madrid Spain; 20 Genetics Unit, Hospital Son Espases, Palma de Mallorca, Balearic Islands, Spain; 21 Genetics Deparment, Hospital Universitario Central de Asturias, Oviedo, Asturias Spain; 22 Department of Ophthalmology, Hospital Universitario de Donostia, San Sebastián, Basque Country Spain; 23 Division of Neurosciences, Biodonostia Health Research Institute, San Sebastián, Basque Country Spain; 24 Department of Ophthalmology, University of the Basque Country-UPV/EHU, Vizcaya, Basque Country Spain; 25 Clinical Genetics Department, Hospital Universitario Miguel Servet (HUMS), Zaragoza, Aragon Spain; 26 Cellular Therapy Service, Blood and Tissue Bank, Building Dr. Frederic Duran I Jordà, Barcelona, Catalonia Spain; 27 Musculoskeletal Tissue Engineering Group, Vall D’Hebron Research Institute (VHIR), Universitat Autònoma de Barcelona, Barcelona, Catalonia Spain; 28 Department of Medical Genetics, Complejo Hospitalario de Navarra (CHN), Pamplona, Navarre Spain; 29 Navarra Institute for Health Research (IdiSNA), Pamplona, Navarre Spain; 30 Molecular Genetics Unit, Hospital Sant Joan de Deu, Barcelona, Catalonia Spain; 31 Molecular (Epi)Genetic Lab, Bioaraba Health Research Institute, Araba University Hospital, Vitoria-Gasteiz, Alava, Basque Country Spain; 32 Clinical Genetics and Functional Genomics Unit, Pediatrics Service, Hospital Clinico Universitario “Lozano Blesa”, Facultad de Medicina, Universidad de Zaragoza, Zaragoza, Aragon Spain; 33 Department of Clinical Analysis, Hospital Universitario General de Valencia, Valencia, Valencian Community Spain; 34 Genetics Unit, Hospital Materno Infantil Teresa Herrera, Hospital Universitario de A Coruña, A Coruña, Galicia, Spain; 35 Instituto Investigación Illes Balears (IDISBA), Palma de Mallorca, Balearic Islands Spain; 36 Department of Ophthalmology, Hospital de la Santa Creu I Sant Pau, Universitat Autònoma de Barcelona, Barcelona, Catalonia Spain; 37 Department of Genetics, Hospital Universitario Donostia, San Sebastian, Basque Country Spain; 38 Genetics Unit, Hospital Universitari Vall D’Hebron, Barcelona, Catalonia Spain; 39 Instituto Oftalmológico Fernández-Vega, Oviedo, Asturias Spain; 40 Department of Ophthalmology, Columbia University, New York, NY USA; 41 Department of Pathology and Cell Biology, Columbia University, New York, NY USA; 42 Medical Genetics, Department of Precision Medicine, Università degli Studi della Campania ‘Luigi Vanvitelli’, Naples, Italy; 43 Telethon Institute of Genetics and Medicine, Pozzuoli, Italy; 44 Department of Human Genetics, Radboud University Medical Center, Nijmegen, The Netherlands; 45 Department of Human Genetics and Donders Institute for Brain, Cognition and Behaviour, Radboud University Medical Center, Nijmegen, The Netherlands; 46 Center for Medical Genetics Ghent (CMGG), Ghent University and Ghent University Hospital, Ghent, Belgium; 47 Center for Applied Genomics, Children’s Hospital of Philadelphia, Philadelphia, PA USA; 48 Division of Human Genetics, Children’s Hospital of Philadelphia, Philadelphia, PA USA; 49 Department of Pediatrics, Perelman School of Medicine, University of Pennsylvania, Philadelphia, PA USA; 50 Institute for Ophthalmic Research, Center for Ophthalmology, University of Tübingen, Tübingen, Germany; 51 Clinical Research Center, Institute of Molecular and Clinical Ophthalmology Basel (IOB), Basel, Switzerland; 52 Department of Ophthalmology, University Hospital Basel, Basel, Switzerland; 53 Department of Genetics and Genome Biology, University of Leicester, Leicester, UK; 54 Department of Ophthalmology, Hadassah Medical Center, Faculty of Medicine, The Hebrew University of Jerusalem, Jerusalem, Israel; 55 Department of Maternofoetal Medicine, Genetics and Reproduction, Institute of Biomedicine of Seville (IBIS), University Hospital Virgen del Rocío-CSIC-University of Seville, Seville, Spain; 56 Department of Biochemistry, Genetics and Immunology, Facultad de Biología, Universidad de Vigo, Pontevedra, Galicia Spain; 57 Research Group on Rare Diseases and Pediatric Medicine, Instituto de Investigacion Sanitaria Galicia Sur (IISGS), Vigo, Galicia Spain; 58 Centro de Investigaciones Biomédicas (CINBIO), Universidad de Vigo, Vigo, Galicia Spain

Correction to: *Scientific Reports* 10.1038/s41598-021-81093-y, published online 15 January 2021

The original version of this Article contained an error, where the corresponding email of Carmen Ayuso was incorrect. Correspondence and requests for materials should be addressed to cayuso@fjd.es

Furthermore, the Article contained errors in affiliations 1, 3, 4 and 5, which were incorrectly given as ‘Department of Genetics, Instituto de Investigación Sanitaria-Hospital Universitario Fundación Jiménez Díaz, Madrid, Spain.’, ‘Department of Ophthalmology, Instituto de Investigación Sanitaria-Hospital Universitario Fundación Jiménez Díaz, Madrid, Spain.’, ‘ Research Group on Molecular, Cellular and Genomical Medicine, Instituto de Investigación Sanitaria La Fe (IIS La Fe), Valencia, Valencian Community, Madrid, Spain.’ and ‘Clinical Genetics Department, Instituto de Investigación Sanitaria Hospital Universitario de la Princesa, Madrid, Spain.’, respectively

The correct affiliations are listed below:

Affiliation 1:

Department of Genetics, Health Research Institute-Fundación Jiménez Díaz University Hospital, Universidad Autónoma de Madrid (IIS-FJD, UAM), Madrid, Spain.

Affiliation 3:

Department of Ophthalmology, Health Research Institute-Fundación Jiménez Díaz University Hospital, Universidad Autónoma de Madrid (IIS-FJD, UAM), Madrid, Spain.

Affiliation 4:

Department of Genetics, Health Research Institute-Fundación Jiménez Díaz University Hospital, Universidad Autónoma de Madrid (IIS-FJD, UAM), Madrid, Spain.

Affiliation 5:

Center for Biomedical Network Research on Rare Diseases (CIBERER), Instituto de Salud Carlos III, Madrid, Spain.

These errors have now been corrected in the HTML and PDF versions of the Article.

