# Supplementary material for: Genetic landscape of 6089 inherited retinal dystrophies affected cases in Spain and their therapeutic and extended epidemiological implications
Source: Sci Rep. 2021 Jan 15;11:1526. doi: 10.1038/s41598-021-81093-y (PMC7810997; doi:10.1038/s41598-021-81093-y)
Supplement: Supplementary file 1 — Supplementary information. [file 41598_2021_81093_MOESM1_ESM.docx]

**Genetic landscape of 6089 inherited retinal dystrophies affected cases in Spain and their therapeutic and extended epidemiological implications**

Irene Perea-Romero, Gema Gordo, Ionut F. Iancu, Marta Del Pozo-Valero, Berta Almoguera, Fiona Blanco-Kelly, Ester Carreño, Belen Jimenez-Rolando, Rosario Lopez-Rodriguez, Isabel Lorda-Sanchez, Inmaculada Martin-Merida, Lucia Pérez de Ayala, Rosa Riveiro-Alvarez, Elvira Rodriguez-Pinilla, Saoud Tahsin-Swafiri, Maria J. Trujillo-Tiebas, the ESRETNET Study Group, the ERDC Study Group, the Associated Clinical Study Group, Blanca Garcia-Sandoval, Pablo Minguez, Almudena Avila-Fernandez, Marta Corton, Carmen Ayuso

**Supplementary Figure S1. Workflow diagram followed along the study.**


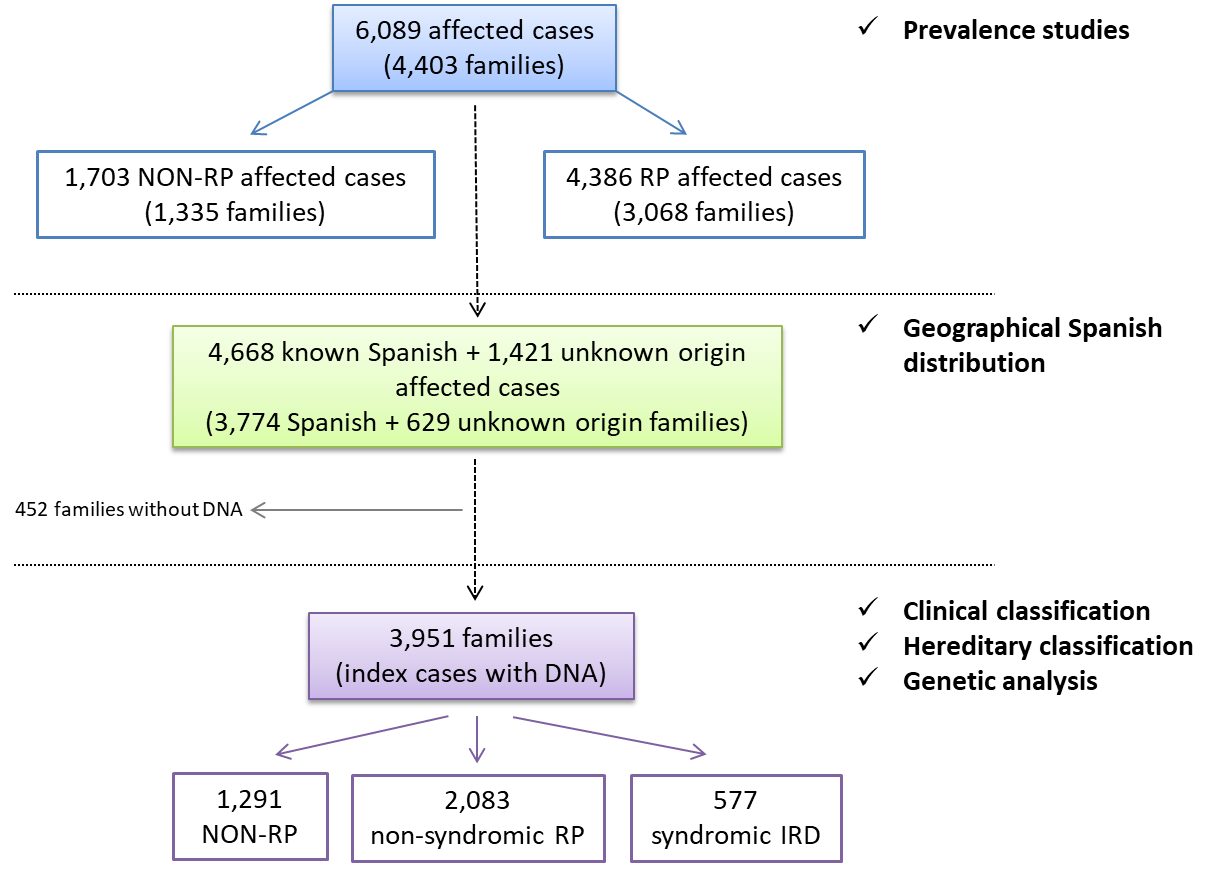


**Supplementary Table S1. Estimated prevalence in the 20%, 53% and 100% of the population of Spain.**

|  | 20% | 53% | 100% |
| --- | --- | --- | --- |
| NON-RP  (N=1.703) | 1:5 487  (1:5 238 - 1:5 761) | 1:14 541  (13 882 - 1:15 266) | 1:27 436  (1:26 192 - 1:28 804) |
| RP  (N=3.561) | 1:2 624  (1:2 541 - 1:2 713) | 1:6 954  (1:6 733 - 1:7 190) | 1:13 121  (1:12 704 - 1:13 566) |
| Syndromic IRD  (N=825) | 1:11 327  (1:10 603 - 1:12 156) | 1:30 016  (1:28 099 - 1:32 214) | 1:56 634  (1:53 016 - 1:60 781) |
| Total RD  (N=6.089) | 1:1 535  (1:1 497 - 1:1 574) | 1:4.067  (1:3 967 - 1:4 172) | 1:7.673  (1:7 485 - 1:7 871) |

Considering a worldwide prevalence of 1:1,000-4,000, our cohort would represent 20-53% of the total patients with IRD in Spain. Confidence intervals between brackets. RD: retinal dystrophies; IRD: inherited retinal dystrophies; RP: retinitis pigmentosa.

**Supplementary Table S2. Comparative table of *a priori* vs final inheritance pattern and reclassification.**

|  | NON-RP | RP | Total |
| --- | --- | --- | --- |
| Inheritance classification without changes | 310 (41.1%) | 555 (53.5%) | 865 (48.3%) |
| *A priori* S – final AR | 378 (50.1%) | 379 (36.5%) | 757 (42.2%) |
| *A priori* unclassified to AD, AR or XL | 11 (1.5%) | 13 (1.2%) | 24 (1.3%) |
| Reclassification (other types) | 55 (7.3%) | 91 (8.8%) | 146 (8.2%) |
| Total | 754 | 1,038 | 1,792 |

Regarding their *a priori* and final inheritance patterns in the NON-RP and RP characterized families, 4 categories have been made: i) families without changes in the inheritance classification (the *a priori* inheritance pattern is the same as the final one), ii) patients with no family history characterized with an AR gene, iii) unclassified families due to the lack of *a priori* family information and iv) properly reclassified families (other types) with a change in the *a priori* inheritance pattern due to the causative gene found after the genetic testing. AD: autosomal dominant; AR: autosomal recessive; S: sporadic.

**Supplementary Table S3. Genes identified in NON-RP group subdivided by mode of inheritance.**

| Nº of families | Gene | Inheritance |
| --- | --- | --- |
| 51 | *PRPH2* | AD |
| 33 | *BEST1* | AD |
| 10 | *GUCY2D* | AD |
| 7 | *CRX* | AD |
| 5 | *PROM1* | AD |
| 4 | *GUCA1A* | AD |
| 2 | *RHO** | AD |
| 2 | *IMPG1* | AD |
| 1 | *AIPL1* | AD |
| 1 | *COL11A1** | AD |
| 1 | *FSCN2** | AD |
| 1 | *IMPG2* | AD |
| 1 | *PRPF31* | AD |
| 1 | *PRPF8** | AD |
| 1 | *TIMP3* | AD |
| 450 | *ABCA4* | AR |
| 21 | *CRB1* | AR |
| 9 | *BEST1* | AR |
| 9 | *CNGB3* | AR |
| 9 | *PROM1* | AR |
| 8 | *CERKL* | AR |
| 5 | *CNGA3* | AR |
| 3 | *USH2A* | AR |
| 3 | *BBS1* | AR |
| 3 | *RDH12* | AR |
| 3 | *RLBP1* | AR |
| 2 | *CDHR1* | AR |
| 2 | *NR2E3* | AR |
| 2 | *RAB28* | AR |
| 1 | *ADAM9* | AR |
| 1 | *AHI1** | AR |
| 1 | *CEP290* | AR |
| 1 | *CDH3** | AR |
| 1 | *CNGB1** | AR |
| 1 | *GNAT2* | AR |
| 1 | *IMPG2* | AR |
| 1 | *KCNV2* | AR |
| 1 | *MYO7A** | AR |
| 1 | *NMNAT1* | AR |
| 1 | *PDE6C* | AR |
| 1 | *RPGRIP1* | AR |
| 1 | *TRPM1** | AR |
| 1 | *CYP4V2* | AR |
| 1 | *TULP1* | AR |
| 83 | *RS1* | XL |
| 2 | *CHM** | XL |
| 2 | *RPGR_ORF15* | XL |
| 1 | *RPGR* | XL |
| 1 | *OPN1LW-OPN1MW* | XL |
| 754 | **Total families** | |

There is a total of 15 genes found in the AD-NON-RP group, 29 in the AR-NON-RP and 4 in the XL-NON-RP. AD: autosomal dominant; AR: autosomal recessive; RP: retinitis pigmentosa; XL: X-linked.

* Clinically reclassified to RP (*RHO, FSCN2, PRPF8, AHI1, CNGB1, TRPM1* and *CHM*) or syndromic IRD (*COL11A1, CDH3* and *MYO7A)* groups.

**Supplementary Table S4. Genes identified in RP group subdivided by mode of inheritance.**

| Nº of families | Gene | Inheritance |
| --- | --- | --- |
| 65 | *RHO* | AD |
| 34 | *PRPF31* | AD |
| 16 | *RP1* | AD |
| 14 | *PRPH2* | AD |
| 12 | *SNRNP200* | AD |
| 11 | *IMPDH1* | AD |
| 9 | *CRX* | AD |
| 9 | *NR2E3* | AD |
| 5 | *PRPF8* | AD |
| 5 | *BEST1* | AD |
| 4 | *C1QTNF5** | AD |
| 4 | *PRPF3* | AD |
| 4 | *TOPORS* | AD |
| 3 | *NRL* | AD |
| 3 | *PRPF6* | AD |
| 2 | *RP1L1* | AD |
| 1 | *FZD4** | AD |
| 1 | *GUCA1A** | AD |
| 1 | *GUCA1B* | AD |
| 1 | *HK1* | AD |
| 1 | *OPA1** | AD |
| 1 | *OPN1SW* | AD |
| 1 | *PROM1* | AD |
| 127 | *USH2A* | AR |
| 56 | *CRB1* | AR |
| 52 | *ABCA4* | AR |
| 37 | *EYS* | AR |
| 28 | *RP1* | AR |
| 27 | *CNGB1* | AR |
| 27 | *CERKL* | AR |
| 26 | *RDH12* | AR |
| 23 | *RPE65* | AR |
| 22 | *NR2E3* | AR |
| 17 | *PDE6A* | AR |
| 15 | *CNGA1* | AR |
| 14 | *CEP290* | AR |
| 11 | *NMNAT1* | AR |
| 10 | *CNGB3** | AR |
| 10 | *TULP1* | AR |
| 9 | *AIPL1* | AR |
| 9 | *MERTK* | AR |
| 9 | *PROM1* | AR |
| 7 | *FAM161A* | AR |
| 7 | *RPGRIP1* | AR |
| 7 | *SPATA7* | AR |
| 6 | *C2orf71* | AR |
| 6 | *LCA5* | AR |
| 6 | *PDE6B* | AR |
| 5 | *PRCD* | AR |
| 5 | *SAG* | AR |
| 4 | *HGSNAT* | AR |
| 4 | *CDHR1* | AR |
| 4 | *CLN3* | AR |
| 4 | *CNGA3** | AR |
| 4 | *GUCY2D* | AR |
| 4 | *LRAT* | AR |
| 4 | *RLBP1* | AR |
| 4 | *RDH5* | AR |
| 3 | *BBS1* | AR |
| 3 | *BBS2* | AR |
| 3 | *IMPG2* | AR |
| 3 | *MAK* | AR |
| 3 | *RP1L1* | AR |
| 2 | *ACBD5** | AR |
| 2 | *GNAT2** | AR |
| 2 | *GRM6* | AR |
| 2 | *IMPG1* | AR |
| 2 | *NRL* | AR |
| 2 | *PDE6C** | AR |
| 2 | *SAMD11* | AR |
| 2 | *SLC24A1* | AR |
| 2 | *TRPM1* | AR |
| 2 | *ZNF408* | AR |
| 2 | *RHO* | AR |
| 1 | *AHI1* | AR |
| 1 | *BEST1* | AR |
| 1 | *ATF6** | AR |
| 1 | *BBS9* | AR |
| 1 | *CLRN1* | AR |
| 1 | *CYP4V2* | AR |
| 1 | *FLVCR1* | AR |
| 1 | *GPR179* | AR |
| 1 | *IFT140* | AR |
| 1 | *KCNV2* | AR |
| 1 | *MYO7A** | AR |
| 1 | *OAT** | AR |
| 1 | *PCDH21* | AR |
| 1 | *PDE6G* | AR |
| 1 | *PDE6H** | AR |
| 1 | *RBP4* | AR |
| 1 | *RGR* | AR |
| 1 | *PRPH2* | AR |
| 1 | *TTPA* | AR |
| 54 | *CHM* | XL |
| 44 | *RPGR_ORF15* | XL |
| 24 | *RPGR* | XL |
| 22 | *RP2* | XL |
| 8 | *CACNA1F* | XL |
| 6 | *NYX* | XL |
| 3 | *OPN1LW-OPN1MW** | XL |
| 2 | *RS1** | XL |
| 1 | *OFD1* | XL |
| 1 | *GPR143** | XL |
| 1,038 | **Total families** | |

There is a total of 23 genes found in the AD-RP group, 70 in the AR-RP and 9 in the XL-RP. AD: autosomal dominant; AR: autosomal recessive; RP: retinitis pigmentosa; XL: X-linked.

* Clinically reclassified to RP (*C1QTNF5, GUCA1A, CNGB3, CNGA3, ACBD5, GNAT2, PDE6C, ATF6, PDE6H, OPN1LW-OPN1MW* and *RS1*) or syndromic IRD (*MYO7A)* groups. Moreover, 4 genes were related to other visual diseases (*FZD4, OPA1, GPR143* and *OAT*).

**Supplementary Table S5. Genes identified in syndromic IRD (USH1, USH2, atypical Usher syndrome and other syndromes).**

| Nº of families | Gene | Disease |
| --- | --- | --- |
| 30 | *MYO7A* | USH1 |
| 12 | *CDH23* | USH1 |
| 9 | *PCDH15* | USH1 |
| 2 | *USH1C* | USH1 |
| 2 | *USH1G* | USH1 |
| 1 | *ADGRV1* | USH1 |
| 129 | *USH2A* | USH2 |
| 11 | *ADGRV1* | USH2 |
| 3 | *MYO7A* | USH2 |
| 1 | *CDH23* | USH2 |
| 1 | *PEX6* | USH2 |
| 5 | *USH2A* | Usher others |
| 1 | *USH3A* | Usher others |
| 23 | *BBS1* | others |
| 6 | *MKKS* | others |
| 5 | *ALMS1* | others |
| 4 | *BBS10* | others |
| 4 | *BBS12* | others |
| 4 | *VPS13B* | others |
| 3 | *NPHP1* | others |
| 3 | *OTX2* | others |
| 2 | *ADNmt* | others |
| 2 | *AHI1* | others |
| 2 | *BBS2* | others |
| 2 | *CEP290* | others |
| 2 | *CWC27* | others |
| 2 | *NPHP5* | others |
| 2 | *PEX1* | others |
| 2 | *RDH12** | others |
| 2 | *SCAPER* | others |
| 1 | *ABHD12* | others |
| 1 | *BBS3* | others |
| 1 | *BBS5* | others |
| 1 | *BBS7* | others |
| 1 | *BBS9* | others |
| 1 | *C8orf37* | others |
| 1 | *CDH23* | others |
| 1 | *CEP41* | others |
| 1 | *CHD7* | others |
| 1 | *CLN8* | others |
| 1 | *COL4A5* | others |
| 1 | *CRB1* | others |
| 1 | *FALDH* | others |
| 1 | *FLVCR1* | others |
| 1 | *HADHA* | others |
| 1 | *IFT140* | others |
| 1 | *IFT27* | others |
| 1 | *INPP5E* | others |
| 1 | *JAG1* | others |
| 1 | *KIF11* | others |
| 1 | *MKS1* | others |
| 1 | *MT-TH* | others |
| 1 | *NPHP4* | others |
| 1 | *PANK2* | others |
| 1 | *PDE6A** | others |
| 1 | *PEX6* | others |
| 1 | *PRPS1* | others |
| 1 | *RPGRIP1** | others |
| 1 | *COL2A1* | others |
| 1 | *SCA7* | others |
| 1 | *WDR19* | others |
| 308 | **Total families** | |

There is a total of 6 genes found in USH1, 5 in USH2, 2 in atypical Usher syndrome and 48 in other syndromes. IRD: inherited retinal dystrophies; USH1: Usher syndrome type 1; USH2: Usher syndrome type 2.

* Clinically reclassified to RP (*RDH12, PDE6A* and *RPGRIP1*).

**Supplementary Table S6. Most frequent causing-variants in NON-RP, RP and syndromic IRD in the Spanish population.**

|  | **Nº of alleles** | **Gene** | **Nucleotide change** | **Amino acid change** | **Reported phenotype** | **Allele frequency: Spanish cohort** | **GnomAD allele frequency: ALL** | **GnomAD allele frequency: Southern European** | **PMID** |
| --- | --- | --- | --- | --- | --- | --- | --- | --- | --- |
| **NON-RP** | 180 | *ABCA4* | c.3386G>T | p.Arg1129Leu | STGD1 | 4.98e-2 | 2.97e-4 | 8.61e-4 | 9295268 |
|  | 62 | *ABCA4* | c.5882G>A | p.Gly1961Glu | STGD1 | 1.71e-2 | 4.56e-3 | 8.18e-3 | 9295268 |
|  | 26 | *ABCA4* | c.5819T>C | p.Leu1940Pro | FF, arMD | 7.19e-3 | 2.12e-5 | 0.000 | 11385708 |
|  | 25 | *ABCA4* | c.3210_3211dupGT | p.Ser1071CysfsTer14 | STGD1 | 6.91e-3 | 1.99e-5 | 1.74e-4 | 9295268 |
|  | 25 | *ABCA4* | c.1804C>T | p.Arg602Trp | STGD1 | 6.91e-3 | 4.39e-5 | 0.000 | 9973280 |
|  | 24 | *ABCA4* | c.4457C>T | p.Pro1486Leu | STGD1 | 6.63e-3 | 1.22e-4 | 1.82e-4 | 9973280 |
|  | 23 | *ABCA4* | c.2888delG | p.Gly963AlafsTer14 | STGD1 | 6.36e-3 | 3.98e-6 | 0.000 | 11385708 |
|  | 20 | *ABCA4* | c.6179T>G | p.Leu2060Arg | CRD | 5.53e-3 | Not found | Not found | 11385708 |
|  | 18 | *ABCA4* | c.5929G>A | p.Gly1977Ser | STGD1 | 4.98e-3 | 7.98e-6 | 0.000 | 9781034 |
|  | 15 | *ABCA4* | c.3322C>T | p.Arg1108Cys | STGD1 | 4.15e-3 | 1.27e-4 | 1.72e-4 | 9973280 |
|  | 14 | *ABCA4* | c.5714+5G>A | p.? | STGD1 | 3.87e-3 | 3.06e-4 | 1.13e-3 | 9466990 |
|  | 14 | *ABCA4* | c.5044_5058del15 | p.Val1682_Val1686del | STGD1 | 3.87e-3 | 2.80e-5 | 0.000 | 9295268 |
|  | 13 | *ABCA4* | c.4918C>T | p.Arg1640Trp | STGD1 | 3.59e-3 | 3.18e-5 | 0.000 | 9973280 |
|  | 13 | *ABCA4* | c.634C>T | p.Arg212Cys | STGD1 | 3.59e-3 | 1.07e-4 | 1.73e-4 | 9503029 |
|  | 12 | *ABCA4* | c.5461-10T>C | p.? | CRD | 3.32e-3 | 2.27e-4 | 0.000 | 15614537 |
|  | 10 | *PRPH2* | c.584G>T | p.Arg195Leu | CACD | 2.76e-3 | 3.98e-6 | 0.000 | 14557183 |
|  | 10 | *ABCA4* | c.3056C>T | p.Thr1019Met | STGD1 | 2.76e-3 | 3.18e-5 | 0.000 | 9973280 |
| **RP** | 106 (138)^a^ | *USH2A* | c.2276G>T | p.Cys759Phe | arRP; Usher II | 3.81e-2^c^ | 9.68e-4 | 2.25e-3 | 10775529 / 26806561 |
|  | 48 | *CERKL* | c.847C>T | p.Arg283Ter | arRP | 1.33e-2 | 3.32e-4 | 8.71e-4 | 14681825 |
|  | 27 | *CNGB1* | c.2957A>T | p.Asn986Ile | adRP | 7.46e-3 | 1.18e-3 | 4.34e-4 | 17564971 |
|  | 24 | *RP1* | c.1625C>G | p.Ser542Ter | arRP, early-onset | 6.63e-3 | 4.25e-5 | 8.65e-5 | 22917891 |
|  | 21 | *RDH12* | c.295C>A | p.Leu99Ile | LCA | 5.8e-3 | 6.01e-5 | 1.72e-4 | 15322982 |
|  | 21 | *CRB1* | c.2843G>A | p.Cys948Tyr | arRP, early-onset | 5.8e-3 | 2.03e-4 | 8.66e-5 | 18055821 |
|  | 18 | *CNGA1* | c.94C>T | p.Arg32Ter | arRP | 4.98e-3 | 7.23e-5 | 2.63e-4 | 12362048 |
|  | 17 | *CNGB3* | c.1148delC | p.Thr383IlefsTer13 | Achr.; CD | 4.7e-3 | 1.75e-3 | 1.47e-3 | 10888875 |
|  | 15 | *NR2E3* | c.932G>A | p.Arg311Gln | ESCS | 4.15e-3 | 3.95e-4 | 4.17e-4 | 10655056 |
|  | 13 | *CRB1* | c.613_619del | p.Ile205AspfsTer13 | LCA | 3.59e-3 | 3.19e-5 | 1.74e-4 | 11231775 |
|  | 12 | *RHO* | c.1040C>T | p.Pro347Leu | adRP | 3.32e-3 | 3.54e-6 | 0.000 | 2215617 / 29847639 |
|  | 10 | *NR2E3* | c.119-2A>C | p.? | ESCS, arRP | 2.76e-3 | 5.26e-4 | 0.000 | 10655056 |
|  | 10 | *FAM161A* | c.1355_1356delCA | p.Thr452SerfsTer3 | arRP | 2.76e-3 | 9.97e-5 | 1.74e-4 | 20705279 |
| **Syndromic IRD** | 51 (60)^a,b^ | *USH2A* | c.2299delG | p.Glu767SerfsTer21 | arRP; Usher II | 1.66e-2^c^ | 7.02e-4 | 5.19-e4 | 9624053 |
|  | 33 (42)^a,b^ | *BBS1* | c.1169T>G | p.Met390Arg | arRP; BBS | 1.16e-2^c^ | 1.57e-3 | 2.50e-3 | 12118255 |
|  | 32 (138)^a^ | *USH2A* | c.2276G>T | p.Cys759Phe | arRP; Usher II | 3.81e-2^c^ | 9.68e-4 | 2.25e-3 | 10775529 / 26806561 |
|  | 11 (20)^a,b^ | *USH2A* | c.9799T>C | p.Cys3267Arg | arRP; Usher II | 5.53e-3^c^ | 7.08e-6 | 0.000 | 17085681 |

AR-MD: autosomal recessive macular dystrophy; CACD: central areolar choroidal dystrophy; STGD1: Stargardt disease 1; FF: fundus flavimaculatus; ACHM: achromatopsia; AD-RP: autosomal dominant retinitis pigmentosa; AR-RP: autosomal recessive retinitis pigmentosa; BBS: Bardet-Biedl syndrome; CD: cone dystrophy; ESCS: enhanced S-cone syndrome; LCA: Leber congenital amaurosis. Total identified alleles: 3,618. Total identified NON-RP alleles: 1,300. Total different NON-RP variants: 458. Total identified non-syndromic RP alleles: 1,716. Total different non-syndromic RP variants: 836 (appearing 40 of them also in syndromic IRD). Total identified syndromic IRD alleles: 602. Total identified syndromic IRD variants: 295 (appearing 40 of them also in non-syndromic RP).

The gnomAD ALL and Southern European frequencies for each allele were extracted from the gnomAD Exomes v2.1.1 dataset.

^a^ These variants can be found both in RP and syndromic IRD and depending on the second variant identified in compound heterozygosis we could observe one of these two phenotypes. The number of alleles in parentheses corresponds to the total of alleles of RP and syndromic IRD together.

^b^ These variants can be found also in non-syndromic RP, but with a lower frequency than 10 alleles.

^c^ These frequencies correspond to the total of alleles independently of the phenotype (non-syndromic or syndromic) of the cases: *USH2A* p.Cys759Phe (n=138 alleles); *USH2A* p.Glu767SerfsTer21 (n=60); *BBS1* p.Met390Arg (n=42); and *USH2A* p.Cys3267Arg (n=20).

**Supplementary Table S7. Comparative table with the different prevalence studies from other countries**.

| Study | Year | Country | Number of included cases | Diseases | Screened genes | Characterized cases | Number of genes found | Techniques |
| --- | --- | --- | --- | --- | --- | --- | --- | --- |
| Bunker *et al*.^1^ | 1984 | Maine (USA) | 226 cases | BBS, RP, USH | N/A | N/A | N/A | N/A |
| Chelva *et al*.^2^ | 1992 | Western Australia | 266 patients + 125 relatives | BBS, LCA, RP, USH | N/A | N/A | N/A | N/A |
| Ziviello *et al*.^3^ | 2005 | Italy | 43 families | adRP | 12 | 28% (12/43) | 5 | Sanger sequencing |
| Eisenberg *et al*.^4^ | 2013 | Miscellanea | 126 patients | CCRD, LCA, RP | 55 | 70% (88/126) | 28 | RD gene panel |
| Bertelsen *et al.*^5^ | 2014 | Denmark | 3,076 cases | IRD | N/A | N/A | N/A | N/A |
| Huang *et al*.^6^ | 2014 | China | 179 families | IRD | 252 | 55.3% (99/179) | 35 | Customized targeted gene panel |
| Beheshtian *et al*.^7^ | 2015 | Iran | 13 families | arRP | 245 | 77% (10/13) | 9 | WES |
| Tiwari *et al*.^8^ | 2016 | Switzerland | 58 patients | IRD | Unknown | 64% (37/58) | 18 | WES |
| Bravo-Gil *et al*.^9^ | 2017 | Spain | 106 cases | sRP | 68 | 62.26% (66/106) | 26 | Customized targeted gene panel |
| Birtel *et al.*^10^ | 2018 | Germany | 251 patients | CCRD, MD | Unknown | 74% (185/251) | 22 | Sanger, MLPA, NGS-panel |
| Maeda *et al*.^11^ | 2018 | Japan | 94 probands | CCRD, RP, BCR, USH, STGD | 39 | 47.9% (45/94) | 19 | Customized targeted gene panel |
| Motta *et al*.^12^ | 2018 | Brazil | 1,246 patients (1,159 families) | IRD | Unknown | 71.6% (400/559) | 66 | aCGH, APEX, WES, NGS-panel, Sanger |
| Kim *et al*.^13^ | 2019 | South Korea | 86 cases | IRD | 204 | 44.2% (38/86) | 22 | Customized targeted gene panel |
| Holtan *et al*.^14^ | 2020 | Norway | 866 patients | IRD | Unknown | 32% (207/650) | 56 | APEX. HTS panel |
| Sharon *et al*.^15^ | 2019 | Israel | 3,413 cases (2,420 families) | IRD | Unknown | 56% (1,369/2,420) | 129 | Several |
| Pontikos *et al*.^16^ | 2020 | United Kingdom | 4,236 patients (3,195 families) | IRD | Unknown | N/A | 135 | Single-gene and gene-panel testing, WES, WGS |
| Goetz *et al*.^17^ | 2020 | USA and Canada | 6,403 cases (5,385 families) | IRD, eye diseases | >200 | 62.1% (3,448/5,552) | Unknown | Several |
| Jaffal *et al*.^18^ | 2020 | 11 Arab countries | 407 individuals from 30 studies  (meta-analysis) | sRCD, arRDC | 63 | N/A | 33 | NGS, homozygosity mapping, Sanger |
| This work | - | Spain | 6,089 cases (4,403 families) | IRD | 291 | 53.2% (2,100/3,951) | 142 | Several^a^ |

adRP: autosomal dominant retinitis pigmentosa; APEX: Arrayer Primer Extension; arRCD: autosomal recessive rod-cone dystrophy; arRP: autosomal recessive retinitis pigmentosa; BBS: Bardet-Biedl syndrome; BCR: Bietti crystallin retinopathy; CCRD: cone/cone-rod dystrophies; HTS: high-throughput sequencing; IRD: inherited retinal dystrophies; LCA: Leber congenital amaurosis; MD: macular dystrophy; MLPA: Multiplex Ligation-dependent Probe Amplification; NGS: Next Generation Sequencing; RD: retinal dystrophies; RP: retinitis pigmentosa; sRCD: sporadic rod-cone dystrophy; sRP: sporadic retinitis pigmentosa; STGD: Stargardt disease; USH: Usher syndrome; WES: Whole Exome Sequencing; WGS: Whole Genome Sequencing.

^a^ All the different techniques used are described in the Supplementary Table S8.

**Supplementary Table S8. Summary table with all the different molecular approaches that have been used during the 28 years of styudy, divided by NON-RP, RP and syndromic IRD and their *a priori* mode of inheritance.**

|  | **Type** | **Technologies** |
| --- | --- | --- |
| **NON-RP** | AD | - Denaturing HPLC - High resolution melting scanning - Sanger sequencing - Haplotype analysis - Customized targeted gene panel - Commercial clinical exome - WES |
|  | AR^19-23^ | - Commercial APEX-based genotyping microarrays (ABCR400 microarray) - Denaturing HPLC - High resolution melting scanning - Sanger sequencing - Customized targeted gene panel - Commercial clinical exome - WES - smMIPS - MLPA - aCGH - Haplotype analysis |
|  | XL | - Sanger sequencing - Haplotype analysis - Commercial APEX-based genotyping microarrays (xlRP microarray including RPGR-ORF15 sequencing) - Customized targeted gene panel - Commercial clinical exome |
|  | Unclassified^22^ | - Commercial clinical exome - smMIPS |
| **RP** | AD^24-28^ | - SSCP - DGGE - Denaturing HPLC - Sanger sequencing - Commercial APEX-based genotyping microarrays (adRP, xlRP microarray including RPGR-ORF15 sequencing) - Customized targeted gene panel (74 IRD-associated genes) - Commercial clinical exome - WES - MLPA - aCGH - Haplotype analysis |
|  | AR^20,23,29-33^ | - Commercial APEX-based genotyping microarrays (arRP, LCA, ABCR400, xlRP microarray including RPGR-ORF15 sequencing) - Denaturing HPLC - Haplotype analysis - Customized targeted gene panel (75 IRD-associated genes) - Commercial clinical exome - WES - WGS - MLPA |
|  | S^30,34^ | - DGGE - Denaturing HPLC - Commercial APEX-based genotyping microarrays (arRP, LCA, ABCR400 microarray) - Sanger sequencing - Customized targeted gene panels (75 IRD-associated genes and 82 RP-associated genes) - Commercial clinical exome - WES - WGS - MLPA - aCGH |
|  | XL | - Sanger sequencing - DGGE - Commercial APEX-based genotyping microarrays (arRP, adRP, LCA, CSNB, xlRP microarrays including RPGR-ORF15 sequencing) - Haplotype analysis - Customized targeted gene panel - Commercial clinical exome - WES - WGS - MLPA |
|  | Unclassified | - Sanger sequencing - Commercial APEX-based genotyping microarrays (arRP, Usher microarray) - Denaturing HPLC - Haplotype analysis - Customized targeted gene panel - Commercial clinical exome |
| **Syndromic IRD** | Usher I^31,35^ | - Commercial APEX-based genotyping microarrays (Usher syndrome microarray) - Sanger sequencing - Customized targeted gene panel - Commercial clinical exome - MLPA |
|  | Usher II^23,31,35^ | - Commercial APEX-based genotyping microarrays (Usher syndrome microarray) - Sanger sequencing - Customized targeted gene panel - Commercial clinical exome - MLPA |
|  | Usher others | - SSCP - Sanger sequencing - Commercial APEX-based genotyping microarrays (arRP, LCA, Usher syndrome, BBS microarrays) - Customized targeted gene panel - Commercial clinical exome |
|  | Others^36-38^ | - Commercial APEX-based genotyping microarrays (BBS, ALMS, LCA, arRP microarrays) - Sanger sequencing - Customized targeted gene panels - Commercial clinical exome - WES - aCGH - MLPA - Haplotype analysis - Mitochondrial DNA sequencing |

aCGH: Microarray-based Comparative Genomic Hybridization; adRP: autosomal dominant retinitis pigmentosa; ALMS: Alström syndrome; APEX: Arrayed Primer Extension; arRP: autosomal recessive retinitis pigmentosa; BBS: Bardet-Biedl syndrome; CSNB: congenital stationary night blindness; DGGE: CG-clamped denaturing gradient gel electrophoresis; HPLC: High-Performance Liquid Cromatography; IRD: inherited retinal dystrophies; LCA: Leber congenital amaurosis; MLPA: Multiplex Ligation-dependent Probe Amplification; RP: retinitis pigmentosa; smMIPS: single-molecule molecular inversion probes; SSCP: Single-strand conformation polymorphism (SSCP); WES: Whole Exome Sequencing; WGS: Whole Genome Sequencing; xlRP: X-linked retinitis pigmentosa.

**Supplementary Appendix S1. Lists of studied genes**

Different diagnostic technologies were used to study the cases. A maximum of 291 genes were analysed.

In cases of non-syndromic inherited retinal dystrophies (IRD) (NON-RP and RP), the following 190 genes were studied: *ABCA4, ACO2, ADAM9, ADIPOR1, AFG3L2, AGBL5, AHR, AIPL1, ARHGEF18, ARL2BP, ARL3, ARL6, ASRGL1, ATF6, ATL3, BBS1, BBS2, BEST1, C12orf65, C1QTNF5, C21orf2, C2orf71, C8orf37, CA4, CABP4, CACNA1F, CACNA2D4, CAPN5, CCT2, CDH3, CDH3, CDHR1, CEP290, CEP78, CERKL, CFH, CHM, CLCC1, CLRN1, CLUAP1, CNGA1, CNGA3, CNGB1, CNGB3, CNNM4, CRB1, CRX, CTNNA1, CYP4V2, DHDDS, DHX38, DMD, DRAM2, DTHD1, EFEMP1, ELOVL1, ELOVL4, EMC1, EYS, FAM161A, FSCN2, FZD4, GDF6, GNAT1, GNAT2, GNB3, GPR125, GPR143, GPR179, GRK1, GRM6, GUCA1A, GUCA1B, GUCY2D, HGSNAT, HK1, HMCN1, IDH3B, IFT140, IFT172, IFT81, IMPDH1, IMPG1, IMPG2, IQCB1, ITM2B, KCNJ13, KCNV2, KIAA1549, KIZ, KLHL7, LCA5, LRAT, LRIT3, LRP5, MAK, MAPKAPK3, MERTK, MFN2, MFRP, MFSD8, MIR204, MVK, NBAS, NDP, NEK2, NEUROD1, NMNAT1, NR2E3, NR2F1, NRL, NYX, OAT, OFD1, OPA1, OPN1LW, OPN1MW, OPN1SW, OTX2, PCDH21, PCDHB14, PDE6A, PDE6B, PDE6C, PDE6G, PDE6H, PGK1, PITPNM3, PLA2G5, POC1B, POMGNT1, PRCD, PRDM13, PROM1, PRPF3, PRPF31, PRPF4, PRPF6, PRPF8, PRPH2, RAB28, RAX2, RB1, RBP3, RBP4, RCBTB1, RD3, RDH12, RDH5, REEP6, RGR, RGS9, RGS9BP, RHO, RIMS1, RLBP1, ROM1, RP1, RP1L1, RP2, RP9, RPE65, RPGR, RPGRIP1, RS1, RTN4IP1, SAG, SAMD11, SEMA4A, SLC24A1, SLC7A14, SNRNP200, SPATA7, SPP2, TEAD1, TIMM8A, TIMP3, TMEM126A, TOPORS, TRNT1, TRPM1, TSPAN12, TTC8, TTLL5, TULP1, UNC119, USH2A, VCAN, ZNF408, ZNF513.*

In patients with syndromic IRD, the following 127 genes were studied: *ABCC6, ABHD12, ACBD5, ACO2, ADAMTS18, ADGRV1, ADIPOR1, AFG3L2, AHI1, ALMS1, ARL6, ARSG, ATXN7, BBIP1, BBS1, BBS10, BBS12, BBS2, BBS3, BBS4, BBS5, BBS7, BBS9, C8orf37, CC2D2A, CDH23, CEP164, CEP19, CEP250, CEP290, CEP41, CEP78, CHD7, CIB2, CLN3, CLN8, CLRN1, COL11A1, COL2A1, COL4A5, COL9A1, CSPP1, CWC27, DFNB31, ELOVL4, ESPN, EXOSC2, FALDH, FLVCR1, GNPTG, HADHA, HARS, HGSNAT, HMX1, IFT140, IFT172, IFT27, IFT81, INPP5E, INVS, IQCB1, JAG1, KCNJ13, KIF11, KSS, LAMA1, LRP5, LZTFL1, MFN2, MKKS, MKS1, MT-ATP6, MT-TH, MT-TL1, MT-TP, MT-TS2, MYO7A, NPHP1, NPHP3, NPHP4, NPHP5, OFD1, OPA3, PANK2, PAX2, PCDH15, PCYT1A, PDZD7, PEX1, PEX2, PEX6, PEX7, PHYH, PLK4, PNPLA6, POC1B, POC5, PRPS1, RDH11, RPGRIP1L, SCA7, SCAPER, SDCCAG8, SLC25A46, TIMM8A, TMEM216, TMEM237, TREX1, TRIM32, TRNT1, TTC8, TTPA, TUB, TUBGCP4, TUBGCP6, USH1C, USH1G, USH2A, USH3A, VCAN, VPS13B, WDPCP, WDR19, WFS1, ZNF423.*

Some genes were included in both groups because they have been reported in syndromic and non-syndromic forms of IRD.

**References**

1. Bunker, C.H., Berson, E.L., Bromley, W.C., Hayes, R.P. & Roderick, T.H. Prevalence of retinitis pigmentosa in Maine. *Am J Ophthalmol* **97**, 357-65, doi: 10.1016/0002-9394(84)90636-6 (1984).
2. Chelva, E., McLaren, T.L., Kay, S.M., Collins, D.W., Black, J.L. & Candy, D.R. A retinitis pigmentosa register for western Australia. *Aust N Z J Ophthalmol* **20**, 311-7, doi: 10.1111/j.1442-9071.1992.tb00743.x (1992).
3. Ziviello, C. *et al.* Molecular genetics of autosomal dominant retinitis pigmentosa (ADRP): a comprehensive study of 43 Italian families. *J Med Genet* **42**, e47, doi: 10.1136/jmg.2005.031682 (2005).
4. Eisenberger, T. *et al.* Increasing the yield in targeted next-generation sequencing by implicating CNV analysis, non-coding exons and the overall variant load: the example od retinal dystrophies. *PLoS One* **8**, e78496, doi: 10.1371/journal.pone.0078496 (2013).
5. Bertelsen, M., Jensen, H., Bregnhøj, J.F. & Rosenberg, T. Prevalence of generalized retinal dystrophy in Denmark. *Ophthalmic Epidemiol* **21**, 217-23, doi: 10.3109/09286586.2014.929710 (2014).
6. Huang, X.F. *et al.* Genotype-phenotype correlation and mutation spectrum in a large cohort of patients with inherited retinal dystrophy revealed by next-generation sequencing. *Genet Med* **17**, doi: 10.1038/gim.2014.138 (2015).
7. Beheshtian, M. *et al.* Impact of whole exome sequencing among Iranian patients with autosomal recessive retinitis pigmentosa. *Arch Iran Med* **18**, 776-85 (2015).
8. Tiwari, A. *et al.* Next generation sequencing based identification of disease-associated mutations in Swiss patients with retinal dystrophies. *Sci Rep* **6**, 28755, doi: 10.1038/srep28755 (2016).
9. Bravo-Gil, N. *et al.* Unravelling the genetic basis of simplex Retinitis Pigmentosa cases. *Sci Rep* **7**, 41937, doi: 10.1038/srep41937 (2017).
10. Birtel, J. *et al.* Clinical and genetic characteristics of 251 consecutive patients with macular and cone/cone-rod dystrophy. *Sci Rep* **8**, 4824, doi: 10.1038/s41598-018-22096-0 (2018).
11. Maeda, A. *et al.* Development of a molecular diagnostic test for Retinitis Pigmentosa in the Japanese population. *Jpn J Ophthalmol* **62**, 451-457, doi: 10.1007/s10384-018-0601-x (2018).
12. Motta, F.L., Martin, R.P., Filipelli-Silva, R., Vallim Salles, M. & Ferraz Sallum, J.M. Relative frequency of inherited retinal dystrophies in Brazil. *Sci Rep* **8**, 15939, doi: 10.1038/s41598-018-34380-0 (2018).
13. Kim, M.S. *et al.* Genetic Mutation Profiles in Korean Patients with Inherited Retinal Diseases. *J Korean Med Sci* **34**, e161, doi: 10.3346/jkms.2019.34.e161 (2019).
14. Holtan, J.P., Selmer, K.K., Heimdal, K.R. & Bragadóttir, R. Inherited retinal disease in Norway – a characterization of current clinical and genetic knowledge. *Acta Ophthalmol* **98**, 286-295, doi: 10.1111/aos.14218 (2020).
15. Sharon, D. *et al.* A nationwide genetic analysis of inherited retinal diseases in Israel as assessed by the Israeli inherited retinal disease consortium (IIRDC). *Hum Mutat* **41**, 140-149, doi: 10.1002/humu.23903 (2020).
16. Pontikos, N. *et al.* Genetic Basis of Inherited Retinal Disease in a Molecularly Characterized Cohort of More Than 3000 Families from the United Kingdom. *Ophthalmology* **127**, 1384-1394, doi: 10.1016/j.ophtha.2020.04.008 (2020).
17. Goetz, K.E. *et al.* Genetic testing for inherited eye conditions in over 6,000 individuals through the eyeGENE network. *Am J Med Genet C Semin Med Genet* **184**, 828-837, doi: 10.1002/ajmg.c.31843 (2020).
18. Jaffal, L., Joumaa, H., Mrad, Z., Zeitz, C., Audo, I. & El Shamieh, S. The genetics of rod-cone dystrophy in Arab countries: a systematic review. *Eur J Hum Genet*, doi: 10.1038/s41431-020-00754-0 (2020).
19. Riveiro-Alvarez, R. *et al.* Frequency of *ABCA4* mutations in 278 Spanish controls: an insight into the prevalence of autosomal recessive Stargardt disease. *Br J Ophthalmol* **93**, 1359-64, doi: 10.1136/bjo.2008.148155 (2008).
20. Riveiro-Alvarez, R. *et al.* Outcome of *ABCA4* disease-associated alleles in autosomal recessive retinal dystrophies: retrospective analysis in 420 Spanish families. *Ophthalmology* **120**, 1332-7, doi: 10.1016/j.ophtha.2013.04.002 (2013).
21. Del Pozo-Valero, M. *et al.* Expanded Phenotypic Spectrum of Retinopathies Associated with Autosomal Recessive and Dominant Mutations in PROM1. *Am J Ophthalmol* **207**, 204-214, doi: 10.1016/j.ajo.2019.05.014 (2019).
22. Del Pozo-Valero, M. *et al.* Genotype-Phenotype Correlations in a Spanish Cohort of 506 Families With Biallelic ABCA4 Pathogenic Variants. *Am J Ophthalmol* **219**, 195-204, doi: 10.1016/j.ajo.2020.06.027 (2020).
23. Corton, M. *et al.* Exome sequencing of index patients with retinal dystrophies as a tool for molecular diagnosis. *PLoS One* **8**, e65574, doi: 10.1371/journal.pone.0065574 (2013).
24. Martin-Merida, I. *et al.* Toward the Mutational Landscape of Autosomal Dominant Retinitis Pigmentosa: A Comprehensive Analysis of 258 Spanish Families. *Invest Ophthalmol Vis Sci* **59**, 2345-2354, doi:10.1167/iovs.18-23854 (2018).
25. Fernandez-San Jose, P. *et al.* Targeted Next-Generation Sequencing Improves the Diagnosis of Autosomal Dominant Retinitis Pigmentosa in Spanish Patients. *Invest Opthalmol Vis Sci* **56**, 2173-82, doi: 10.1167/iovs.14-16178 (2015).
26. Martin-Merida, I. *et al.* Analysis of the *PRPF31* Gene in Spanish Autosomal Dominant Retinitis Pigmentosa Patients: A Novel Genomic Rearrangement. *Invest Ophthalmol Vis Sci* **58**, 1045-1053, doi: 10.1167/iovs.16-20515 (2017).
27. Blanco-Kelly, F. *et al.* Dominant Retinitis Pigmentosa, p.Gly56Arg Mutation in *NR2E3*: Phenotype in a Large Cohort of 24 Cases. *PLoS One* **11**, e0149473, doi: 10.1371/journal.pone.0149473 (2016).
28. Almoguera, B. *et al.* Application of Whole Exome Sequencing in Six Families with an Initial Diagnosis of Autosomal Dominant Retinitis Pigmentosa: Lessons Learned. *PLoS One* **10**, e0133624, doi: 10.1371/journal.pone.0133624 (2015).
29. Vallespin, E. *et al.* Mutation screening of 299 Spanish families with retinal dystrophies by Leber congenital amaurosis genotyping microarray. *Invest Ophthalmol Vis Sci* **48**, 5653-61, doi: 10.1167/iovs.07-0007 (2007).
30. Perez-Carro, R. *et al.* Panel-based NGS Reveals Novel Pathogenic Mutations in Autosomal Recessive Retinitis Pigmentosa. *Sci Rep* **6**, 19531, doi: 10.1038/srep19531 (2016).
31. Perez-Carro, R. *et al.* Unravelling the pathogenic role and genotype-phenotype correlation of the USH2A p.(Cys759Phe) variant among Spanish families. *PLoS One* **13**, e0199048, doi: 10.1371/journal.pone.0199048 (2018).
32. Avila-Fernandez, A. *et al.* Mutation analysis of 272 Spanish families affected by autosomal recessive retinitis pigmentosa using a genotyping microarray. *Mol Vis* **16**, 2550-8 (2010).
33. Avila-Fernandez, A. *et al.* Whole-exome sequencing reveals ZNF408 as a new gene associated with autosomal recessive retinitis pigmentosa with vitreal alterations. *Hum Mol Genet* **24**, 4037-48, doi: 10.1093/hmg/ddv140 (2015).
34. Martin-Merida, I. *et al.* Genomic Landscape of Sporadic Retinitis Pigmentosa: Findings from 877 Spanish Cases. *Ophthalmology* **126**, 1181-1188, doi: 10.1016/j.ophtha.2019.03.018 (2019).
35. Blanco-Kelly, F. *et al.* Clinical aspects of Usher syndrome and the USH2A gene in a cohort of 433 patients. *JAMA Ophthalmol* **133**, 157-64, doi: 10.1001/jamaophthalmol.2014.4498 (2015).
36. Sanchez-Navarro, I. *et al.* Combining targeted panel-based resequencing and copy-number variation analysis for the diagnosis of inherited syndromic retinopathies and associated ciliopathies. *Sci Rep* **8**, 5285, doi: 10.1038/s41598-018-23520-1 (2018).
37. Almoguera, B. *et al.* Expanding the phenotype of PRPS1 syndromes in females: neuropathy, hearing loss and retinopathy. *Orphanet J Rare Dis* **9**, 190, doi: 10.1186/s13023-014-0190-9 (2014).
38. Tatour, Y. *et al.* Mutations in *SCAPER* cause autosomal recessive retinitis pigmentosa with intellectual disability. *J Med Genet* **54**, 698-704, doi: 10.1136/jmedgenet-2017-104632 (2017).
